# Omega-3 fatty acids reprogram tumor-associated macrophage cytokine signaling and suppress pancreatic cancer growth

**DOI:** 10.1073/pnas.2618288123

**Published:** 2026-10-16

**Authors:** Stephen Sakuma, Steven Zhao, Andrew Hildreth, Gloria Liu, Valisha Shah, Ming Sun, Shirong Tan, Emily Jacobs, Michael A. LaPorta, Jesse Furguiele, Natalia Avina-Ochoa, Anna-Maria Globig, Victoria Tripple, ImmgenT Consortium, Geraldine Maier, Satchidananda Panda, Michelle L. Reyzer, Jing X. Kang, David W. L. Ma, Matthew Rodeheffer, Saame Raza Shaikh, Susan M. Kaech

**Affiliations:** ^a^ NOMIS Center for Immunobiology and Microbial Pathogenesis, Salk Institute for Biological Studies, La Jolla, CA 92037; ^b^ Regulatory Biology Laboratory, Salk Institute for Biological Studies, La Jolla, CA 92037; ^c^ Department of Biochemistry, Mass Spectrometry Research Center, Vanderbilt University, Nashville, TN 37232; ^d^ Department of Medicine, Laboratory for Lipid Medicine and Technology, Massachusetts General Hospital and Harvard Medical School, Boston, MA 02129; ^e^Omega-3 and Global Health Institute, Boston, MA 02129; ^f^ Department of Human Health and Nutritional Sciences, University of Guelph, Guelph, ON N1G 2W1, Canada; ^g^Department of Comparative Medicine, Yale School of Medicine, New Haven, CT 06520; ^h^Department of Cellular and Molecular Physiology, Yale School of Medicine, New Haven, CT 06520; ^i^ Department of Nutrition, Gillings School of Global Public Health and School of Medicine, University of North Carolina at Chapel Hill, Chapel Hill, NC 27599

**Keywords:** PDAC, tumor-associated macrophages, omega-3 fatty acids, tumor microenvironment, cytokine signaling

## Abstract

Dietary polyunsaturated fatty acids (PUFAs) shape inflammatory signaling within the pancreatic tumor microenvironment (TME), but the cellular mechanisms underlying their effects on tumor progression remain unclear. Using orthotopic models of pancreatic ductal adenocarcinoma (PDAC), we show that omega-3 (n-3) and omega-6 (n-6) PUFAs are differentially incorporated into tumor lipid pools, generating spatially heterogeneous lipid microenvironments. While an n-6–enriched diet accelerated PDAC growth, an n-3–enriched diet suppressed tumor growth in a T cell-independent manner. Examining inflammatory effects in PDAC tumors, the n-3 PUFA–enriched diet reduced pro-inflammatory cytokines, including IL-6 family members, and single-cell and spatial analyses identified tumor-associated macrophages (TAMs) as the primary diet-responsive population. Indeed, selective conversion of n-6 to n-3 fatty acids within myeloid cells using an inducible *fat-1* transgene, which encodes an n-3 desaturase that converts n-6 to n-3 PUFAs in vivo, was sufficient to suppress tumor growth, whereas fibroblast-restricted conversion had no effect. This finding is the first to formally establish that cellular origin of PUFA metabolism within the TME is a key determinant of tumor progression and identifies TAMs as critical mediators of n-3–dependent tumor suppression. Furthermore, the n-3 PUFA–enriched diet reduced Arg1^+^ immunosuppressive TAMs and promoted C1q^+^ and MHC-II^+^ antigen-presenting TAM populations enriched for antigen processing machinery and T cell–recruiting chemokines, alongside reduced IL-6 family cytokine production. Together, these findings establish macrophages as key mediators of n-3–driven tumor control and define a mechanism by which dietary lipids remodel cytokine signaling in the PDAC microenvironment.

## Significance

Dietary omega-3 fatty acids have long been associated with reduced cancer progression, but the mechanisms linking lipid composition to tumor immunity remain poorly understood. Using orthotopic pancreatic cancer models, lipidomics, spatial imaging, and single-cell transcriptomics, we show that omega-3 fatty acids reshape the pancreatic tumor microenvironment (TME) by suppressing IL-6–associated inflammatory programs and promoting interferon-associated and antigen-presenting macrophage states. Remarkably, selective conversion of omega-6 to omega-3 fatty acids within myeloid cells alone was sufficient to suppress tumor growth, whereas fibroblast-specific conversion had no effect. These findings identify macrophages as key mediators of omega-3–dependent tumor suppression and establish that the cellular source of lipid signaling within the TME critically influences pancreatic cancer progression.

Polyunsaturated fatty acids (PUFAs) are essential lipid species that exert broad effects on metabolism, inflammation, and tissue homeostasis (1). The two dominant classes in mammalian biology are omega-6 (n-6) and omega-3 (n-3) PUFAs (1, 2), which are defined by the position of the terminal double bond. While other animals have n-3 desaturases and are able to convert n-6 to n-3 PUFAs, mammalian cells lack these enzymes and are unable to interconvert between these two fatty acid families (3). Therefore, dietary intake dictates the level of n-3 and n-6 PUFAs in humans (4). Omega-6 PUFAs are enriched in plant-based oils common in modern diets, such as soybean, corn, and sunflower oils, with linoleic acid (LA) serving as the major dietary precursor for arachidonic acid (AA) (5, 6). In contrast, omega-3 PUFAs are obtained predominantly from marine sources, with eicosapentanoic acid (EPA) and docosahexaenoic acid (DHA) enriched in fatty fish and fish oils, and α-linolenic acid (ALA) contributing a plant-derived source with limited conversion efficiency to the longer EPA and DHA PUFAs (6–8).

Contemporary Western diets are characterized by disproportionately high intake of omega-6 relative to omega-3 PUFAs, resulting in an elevated n-6:n-3 ratio associated with chronic inflammatory and metabolic diseases (2, 5). Low n-6 to n-3 ratios are consistently associated with reduced cardiovascular risk, including lower rates of coronary heart disease and cardiac mortality, and have also been linked in large cohort studies to reduced risk of cognitive decline and dementia, as well as lower all-cause mortality (9, 10). In addition to these outcomes, omega-3 PUFAs have well-established anti-inflammatory effects and are clinically approved for lowering triglycerides and are associated with improvements in blood pressure and metabolic health, and may contribute to benefits in conditions such as non-alcoholic fatty liver disease, pre-term birth, and systemic inflammatory disorders (11–15).

Beyond their structural roles in membranes, PUFAs are enzymatically converted into lipid mediators that regulate inflammation and immunity (16–18). While n-6–derived eicosanoids such as prostaglandins and leukotrienes are classically associated with pro-inflammatory signaling, n-3 PUFAs give rise to monohydroxylated derivatives and downstream specialized pro-resolving mediators (SPMs), including resolvins, protectins, and maresins, which actively dampen inflammation and promote tissue repair (19). The low abundance, analytical challenges, and transcellular biosynthesis of SPMs have complicated efforts to understand the lipid signals responsible for n-3 PUFA–dependent effects in vivo (20–22), but SPMs have emerged as central regulators of immune cell behavior, influencing dendritic cell and macrophage activation states, cytokine production, and stromal–immune crosstalk (23–25). These properties have raised substantial interest in n-3 PUFAs as modulators of the tumor microenvironment (TME), particularly in cancers characterized by chronic inflammation (26–28).

A growing body of preclinical literature has reported that dietary n-3 PUFAs reduce tumor burden across multiple cancer types, including pancreatic ductal adenocarcinoma (PDAC) (28–31). This is particularly relevant given that obesity and high-fat diet consumption are well-established risk factors for PDAC development and progression, linking dietary lipid exposure to disease pathogenesis (32, 33). In PDAC models, high dietary n-6 PUFAs have been associated with accelerated tumor growth and worse outcomes, whereas n-3 PUFA–enriched diets consistently suppress tumor progression (30, 31). These effects have been accompanied by reduced proliferation and increased apoptosis of tumor cells, phenotypes observed across diverse experimental systems (30, 31, 34). However, despite the reproducibility of these outcomes, mechanistic insight remains limited. Most studies rely on bulk tumor readouts or correlative associations with inflammatory markers, leaving unresolved whether n-3 PUFAs act directly on tumor cells, indirectly through stromal or immune compartments, or via systemic metabolic effects.

Obesity is a major risk factor for PDAC and is tightly linked to chronic inflammation and altered systemic lipid metabolism, raising the possibility that dietary lipids directly shape the TME and influence disease progression (32, 35, 36). PDAC is notable for its dense, inflammatory stroma and profound resistance to immunotherapy, suggesting that lipid composition may regulate tumor–immune interactions in ways that are not yet understood (37–39). While n-3 PUFAs and their metabolites are widely assumed to exert anti-inflammatory effects (16, 17, 40), several key questions remain unresolved: how do dietary lipids alter the TME; which cell types within the tumor sense and respond to these lipids; are changes in the TME immune cell composition, activation state, or spatial organization required for tumor control; and how are lipid signals integrated into inflammatory pathways and cytokine signaling in the TME? Addressing these questions is necessary to move beyond correlative observations and define the mechanisms by which dietary fatty acids influence PDAC biology.

## Results

### Dietary Omega-3 and Omega-6 PUFAs Have Heterogeneous Spatial Localization in the PDAC TME.

**Fig. 1.**
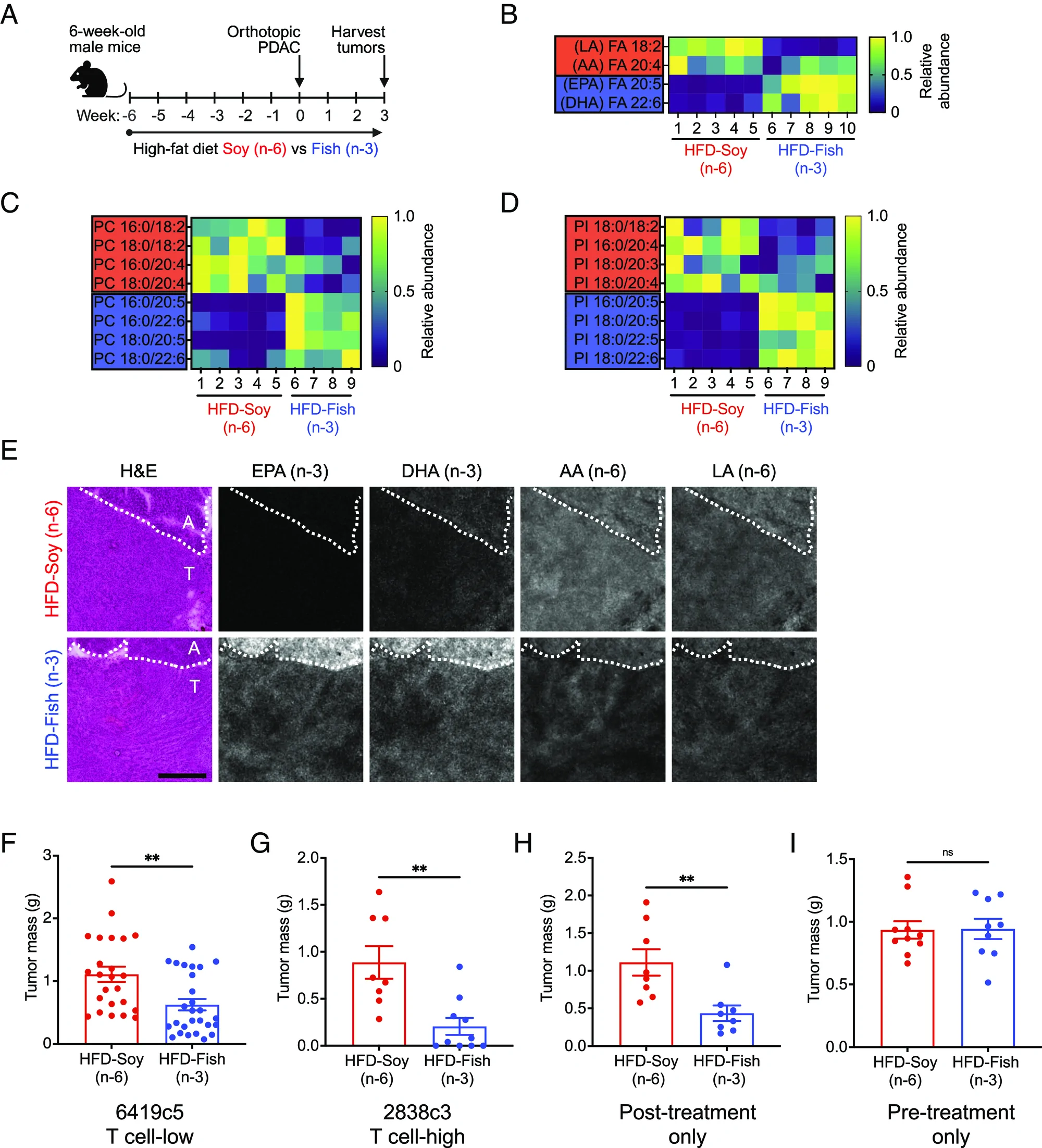
Dietary omega-3 and omega-6 fatty acids generate distinct lipid microenvironments and differentially regulate PDAC tumor growth. (*A*) Experimental schematic of n-6 and n-3 HFD conditioning and orthotopic PDAC implantation. (*B*) LC/MS quantification of free fatty acid (FFA) species in tumor interstitial fluid (TIF) isolated from orthotopic tumors from mice maintained on the indicated diets. (*C*) LC/MS analysis of phosphatidylcholine (PC) species in tumor lysates from mice maintained on the indicated diets. Data are shown as relative abundance normalized to internal standards. (*D*) LC/MS analysis of phosphatidylinositol (PI) species in tumor lysates from mice maintained on the indicated diets. Data are shown as relative abundance normalized to internal standards. (*E*) MALDI-mass spectrometry imaging (MALDI-MSI) of linoleic acid (LA; 18:2, n-6), arachidonic acid (AA; 20:4, n-6), EPA (20:5, n-3), and DHA (22:6, n-3) in orthotopic PDAC tumors from mice maintained on the indicated diets. Dotted line indicates the border between the tumor (T) and normal adjacent tissue (A). Representative from n = 4 biologically independent tumors per group. (Scale bar, 0.5 mm.) (*F*) Endpoint tumor mass of orthotopic 6419c5 tumors harvested at day 19 from mice maintained on the indicated diets. (*G*) Endpoint tumor mass of orthotopic 2838c3 tumors harvested at day 38 from mice maintained on the indicated diets. (*H*) Endpoint tumor mass of orthotopic 6419c5 PDAC tumors from mice receiving post-treatment dietary intervention. (*I*) Endpoint tumor mass of orthotopic 6419c5 PDAC tumors from mice receiving dietary pre-treatment followed by standard chow during tumor progression. Representative images and data from two (*B*–*E*, *H*, and *I*) or three (*F* and *G*) independent experiments. Data are presented as mean ± SEM. Each dot represents one biologically independent mouse. Statistical analysis was performed using Welch’s *t* test (*F*–*I*). *P < 0.05, **P < 0.01, ***P < 0.001, ****P < 0.0001.

Given the strong association between dietary polyunsaturated fatty acid (PUFA) composition and inflammation, and prior evidence that omega-3 PUFAs suppress tumor growth in PDAC, we sought to define how dietary lipid source shapes the TME and alters tumor progression in vivo using a controlled orthotopic PDAC model (Fig. 1*A*). High-fat diets (HFD) were compounded with equivalent composition except for fat sources allowing for tightly controlled mechanistic experiments. In the omega-6 enriched diet, HFD-Soy (n-6), the main fat source is soybean oil while in the omega-3 enriched diet, HFD-Fish (n-3), the main fat source is menhaden fish oil (*SI Appendix*, Fig. S1*A*). The carbohydrate, protein, and fat levels of the diets were equivalent (*SI Appendix*, Fig. S1*B*), but a lipidomic analysis by liquid chromatography–mass spectrometry (LC/MS) of the diet confirmed that the HFD-Fish (n-3) diet contained high levels of EPA and DHA and moderate levels of LA while the HFD-Soy (n-6) diet had high levels of LA and almost no EPA and DHA (*SI Appendix*, Fig. S1*C*). Male mice were conditioned on HFD-Soy (n-6) or HFD-Fish (n-3) diets for 6 wk prior to serum lipidomic analysis, which confirmed corresponding increases in systemic omega-6 and omega-3 PUFA levels (*SI Appendix*, Fig. S1*D*). Together, these diets enabled selective manipulation of systemic PUFA composition while maintaining equivalent caloric and macronutrient intake, allowing the direct in vivo comparison of the biological consequences of omega-6– versus omega-3–enriched lipid environments.

To exclude the possibility that dietary lipid composition induces pancreatic injury or inflammation that could confound tumor growth, we assessed markers of pancreatitis in mice maintained on each diet. No differences were observed in pancreas weight, serum amylase, or serum lipase between diet groups (*SI Appendix*, Fig. S1 *E*–*G*). Furthermore, histological analysis by H&E staining revealed no evidence of tissue damage or inflammatory infiltration, and trichrome staining showed no differences in fibrosis (*SI Appendix*, Fig. S1*H*). Together, these data indicate that the dietary interventions do not induce pancreatitis or baseline pancreatic remodeling, supporting the conclusion that subsequent tumor phenotypes arise from effects within the TME rather than pre-existing tissue pathology.

Using this system, we next tested how dietary PUFA source influences PDAC growth and the TME in an orthotopic transplantation model. To model the immune-excluded state typical of most PDAC, we used the 6419c5 tumor cell line (41) derived from tumors of mice with mutant Kras and p53 (KPC), which recapitulates major features of human PDAC and forms ‘cold’ tumors with low T cell infiltration, a hallmark feature of PDAC tumors that are frequently characterized by poor intratumoral T cell infiltration and an immunosuppressive microenvironment (37, 42). After 6 wk of diet conditioning, pancreatic tumor cells were orthotopically implanted, and tumors were allowed to grow for 3 wk, with each cohort maintained on their respective diets. (Fig. 1*A*). Tumors were harvested and TIF was isolated for lipidomic analysis. First, we compared the relative amounts of FFA in the TIF and, as predicted, observed that tumors from mice on the HFD-Soy (n-6) diet contained elevated omega-6 and reduced omega-3 FFA levels, whereas TIF from tumors of mice on the HFD-Fish (n-3) diet showed the opposite pattern, with increased omega-3 and decreased omega-6 FFA levels (Fig. 1*B*). Next, we analyzed tumor lysates by phospholipidomics to assess incorporation of omega-3 and omega-6 PUFAs into membrane phospholipids, reflecting both their integration into structural lipid bilayers and their entry into signaling-relevant lipid pools. Phosphatidylcholine (PC), the dominant structural membrane phospholipid, showed clear diet-dependent remodeling, with tumors from mice on the HFD-Soy (n-6) diet enriched for omega-6 and depleted of omega-3 PC species, while the opposite pattern was observed in tumors from mice on the HFD-Fish (n-3) diet (Fig. 1*C*). We next examined phosphatidylinositol (PI), a lower-abundance phospholipid that serves as a precursor for phosphoinositide signaling. As with PC, PI composition in tumors from mice on the HFD-Soy (n-6) contained increased omega-6 and reduced omega-3 PI species, with reciprocal changes observed in tumors from mice on the HFD-Fish (n-3) diet (Fig. 1*D*). Together, these data demonstrate that dietary PUFAs remodel the tumor lipid environment across circulating FFAs, structural membrane phospholipids, and signaling-relevant lipid pools.

To determine how dietary fatty acids were spatially distributed in the context of PDAC, we performed mass spectrometry imaging (MSI), which, unlike conventional LC/MS that analyzes bulk tissue extracts, enables spatially resolved mapping of lipid species directly within intact tissue sections. Consistent with the LC/MS data, MSI revealed that tumors from mice fed the HFD-Fish (n-3) diet contained increased levels of omega-3 FFAs, whereas tumors from mice fed the HFD-Soy (n-6) diet showed increased omega-6 FFAs (Fig. 1*E* and *SI Appendix*, Fig. S2*A*). In agreement with the bulk phospholipidomics, mice fed the HFD-Fish (n-3) diet contained increased levels of PI species that contained omega-3, whereas tumors from mice fed the HFD-Soy (n-6) diet contained increased levels of PI species that contained omega-6 (*SI Appendix*, Fig. S2*A*). Notably, the fatty acid landscape within tumors was highly heterogeneous across spatial regions, and spatial analysis further revealed that the omega-3 fatty acids DHA and EPA were less abundant in tumoral regions compared with adjacent normal pancreatic tissue (Fig. 1*E*). Together, these data demonstrate that dietary PUFAs are incorporated into both structural and signaling phospholipid pools within PDAC tumors, generating a spatially heterogeneous lipid microenvironment that reflects dietary lipid source but differs between tumor and adjacent normal tissue, indicating that the tumor has a lipid composition that is distinct from the surrounding tissue.

### Shifting Dietary Omega-6 to Omega-3 Fatty Acids Reduces PDAC Tumor Burden.

We next examined whether dietary PUFA composition altered tumor progression in vivo. Longitudinal ultrasound measurements revealed that 6419c5 tumors, a T cell–low model (41), grew more slowly in mice maintained on the HFD-Fish (n-3) diet compared with those on the HFD-Soy (n-6) diet (*SI Appendix*, Fig. S2*B*), and at endpoint, tumors from mice on the HFD-Fish (n-3) diet displayed significantly reduced tumor mass (Fig. 1*F*). The 6419c5 cell line was derived from prior work demonstrating that individual PDAC clones can generate distinct immunogenic states through tumor cell–intrinsic factors, resulting in tumors with either high or low T cell infiltration (41). We next tested a second model, 2838c3, which was isolated from the same tumor but exhibits a T cell–high phenotype. Consistent with the findings in 6419c5 tumors, mice bearing the T cell–high 2838c3 tumors also exhibited a significant reduction in tumor mass when maintained on the HFD-Fish (n-3) diet (Fig. 1*G*), and similar effects were observed in the FC1199 tumor model which is an additional independently generated KPC mouse tumor (*SI Appendix*, Fig. S2*C*). To test the potency of the HFD-Fish (n-3) diet’s ability to blunt tumor growth, we tested a mixed HFD-Mix (n-3/6) diet containing 50% fish and 50% soybean fat sources. This diet with half of the omega-3 fatty acids still resulted in decreased tumor mass compared with the HFD-Soy (n-6) diet (*SI Appendix*, Fig. S2*D*). Surprisingly, the tumor-suppressive effect of the HFD-Fish (n-3) diet was not observed in female mice (*SI Appendix*, Fig. S2*E*). These results demonstrate that tumor growth is potently suppressed in male mice by the HFD-Fish (n-3) diet across models and that the tumor suppression phenotype is not strictly dependent on T cell abundance, is not blocked by the presence of higher levels of omega-6 fatty acids.

To determine whether differences in tumor growth were driven by systemic metabolic changes, we assessed body weight and metabolic parameters in mice. Mice fed the HFD-Soy (n-6) diet exhibited greater weight gain over the course of the experiment and at tumor harvest (*SI Appendix*, Fig. S2 *F*–*G*), yet metabolic cage analysis revealed no differences in food intake or oxygen consumption between diet groups (*SI Appendix*, Fig. S2 *H*–*I*), indicating comparable caloric intake and basal metabolism. Importantly, even though mice maintained on the HFD-Soy (n-6) diet had increased weight gain, mice maintained on the HFD-Mix (n-3/6) diet did not show any difference in weight gain when compared to mice on the HFD-Soy (n-6) diet (*SI Appendix*, Fig. S2*J*), suggesting that omega-3 dependent control of tumor growth is independent of weight changes. To directly control for weight differences between the HFD-Fish (n-3) and HFD-Soy (n-6) fed mice, we next separated dietary exposure into pre-treatment only (before tumor implantation) and post-treatment only (after tumor implantation) conditions (*SI Appendix*, Fig. S2*K*). At the time of tumor implantation, mice in the pre-treatment HFD-Fish (n-3) group had gained less weight than those on HFD-Soy (n-6), whereas mice in the post-treatment groups were maintained on standard chow and exhibited equivalent weight prior to tumor implantation (*SI Appendix*, Fig. S2 *G*, *L*). Despite these differences, post-treatment with the HFD-Fish (n-3) diet was sufficient to suppress tumor growth compared with HFD-Soy (n-6) (Fig. 1*H*). In contrast, pre-treatment followed by a switch to standard chow after tumor implantation resulted in no difference in tumor size between groups (Fig. 1*I*). These data indicate that the reduction in tumor growth is not attributable to systemic metabolic differences or increased weight, but instead requires ongoing omega-3 exposure during tumor progression, with the lipid environment at implantation alone insufficient to confer sustained tumor control.

### T Cells Are Not Required for Omega-3 Diet-induced Reduction in PDAC Tumor Size.

**Fig. 2.**
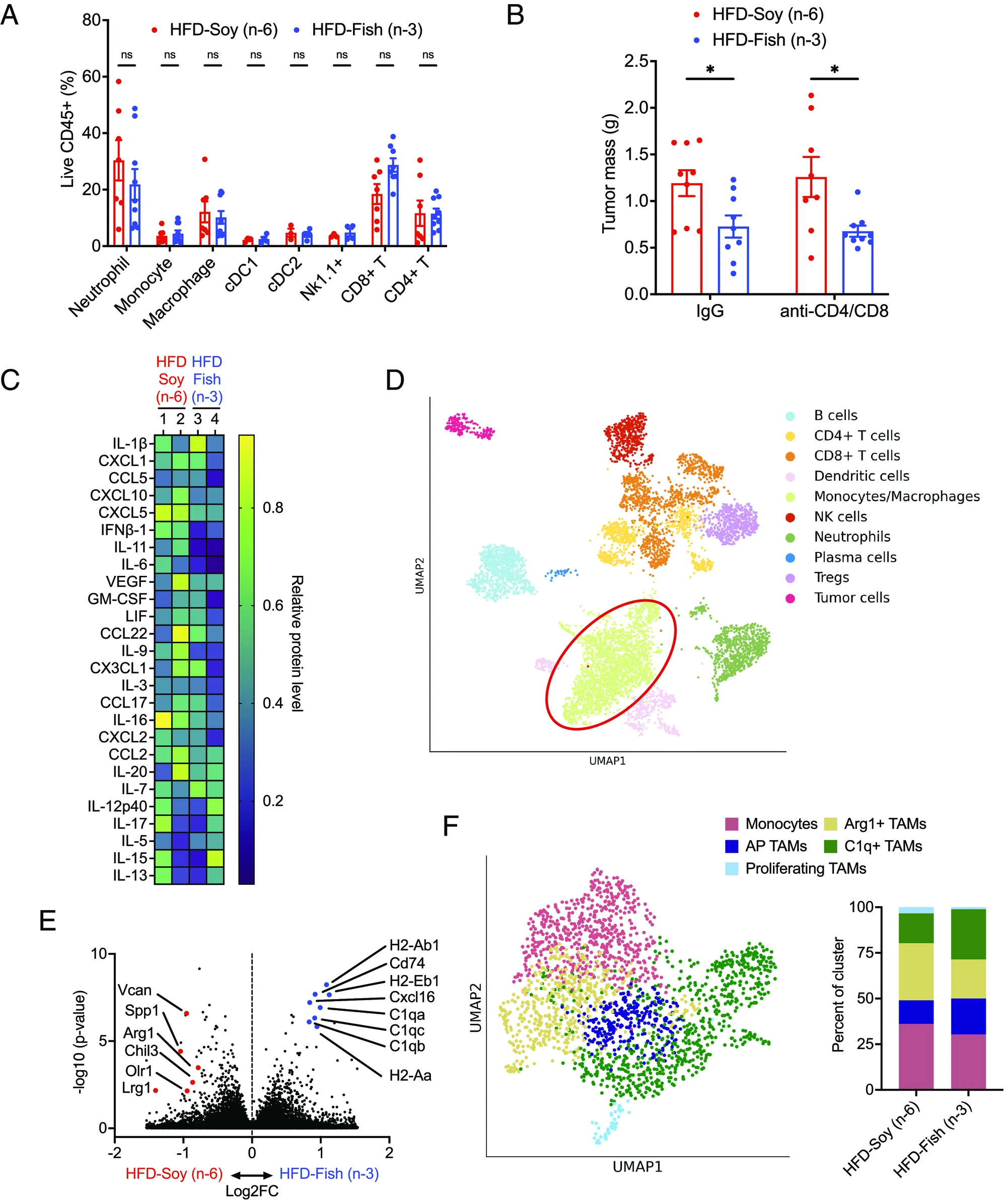
Dietary omega-3 fatty acids alter inflammatory cytokine signaling and macrophage states in the PDAC TME. (*A*) Flow cytometric quantification of immune cell populations in orthotopic 6419c5 tumors from mice maintained on the indicated diets. (*B*) Endpoint tumor mass of orthotopic 6419c5 tumors from mice maintained on the indicated diets following treatment with isotype control antibodies or anti-CD4 and anti-CD8 depleting antibodies. (*C*) Multiplex cytokine array analysis of tumor lysates from orthotopic 6419c5 tumors from mice maintained on the indicated diets. (*D*) UMAP visualization of single-cell RNA sequencing (scRNA-seq) data annotated from sorted CD45^+^CD3^+^ T cells, CD45^+^CD3^–^ leukocytes, and CD45^–^YFP^+^ tumor cells isolated from orthotopic 6419c5 tumors from mice maintained on the indicated diets. (*E*) Volcano plot of differentially expressed genes in the monocyte/macrophage population identified in (*D*) comparing the indicated diets. (*F*) Reclustering analysis of the monocyte/macrophage populations identified in (*D*) with monocytes, *Arg1*^+^ tumor-associated macrophages (TAMs), antigen-presenting TAMs (AP-TAMs), *C1q*^+^ TAMs, and proliferating TAMs subsets. Representative data from two (*A*–*C*) or one (*D*–*F*) independent experiments. For scRNA-seq experiments, two tumors were pooled per sample with n = 2 samples per condition. Data are presented as mean ± SEM. Each dot represents one biologically independent mouse (*A* and *B*). Statistical analysis was performed using two-way ANOVA with Šídák’s multiple comparisons test (*A* and *B*). ns, not significant, *P < 0.05, **P < 0.01, ***P < 0.001, ****P < 0.0001.

To determine whether dietary PUFA composition alters immune features within the TME, we next performed flow cytometry-based immune profiling to identify the major lymphoid and myeloid populations. Comparison between mice fed the HFD-Soy (n-6) diet and the HFD-Fish (n-3) diet did not reveal significant differences in the abundance of these immune subsets. Although there appeared to be a modest trend toward increased CD8^+^ T cells in tumors from mice on the HFD-Fish (n-3) diet (Fig. 2*A*), further analysis of markers associated with CD8^+^ T cell activation and exhaustion showed no detectable differences between the diets (*SI Appendix*, Fig. S3*A*). Because CD8^+^ T cells represent an obvious candidate population that could mediate the observed reduction in tumor burden, we next tested whether T cells were required for this phenotype. Mice were treated with depleting antibodies targeting CD4^+^ and CD8^+^ T cells 1 wk before tumor implantation and throughout the tumor growth period, which efficiently eliminated these populations as confirmed by flow cytometry (*SI Appendix*, Fig. S3*B*). This revealed that the PDAC tumors grew more slowly in mice fed HFD-Fish (n-3) diets regardless of whether the CD4^+^ and CD8^+^ T cells were depleted or not (Fig. 2*B*). These data indicate that the tumor-suppressive effects of dietary omega-3 PUFAs occur independently of T cells, suggesting that other physiological factors in the TME mediate this phenotype.

### Shifting Dietary Omega-6 to Omega-3 Fatty Acids Alters TME Inflammatory Cytokine Tone.

We next sought to identify T cell-independent changes within the TME that might contribute to the slower tumor progression observed with the HFD-Fish diet. Given the established role of PUFAs in regulating inflammatory signaling, we hypothesized that dietary fatty acid composition alters cytokine and chemokine production within the PDAC TME. To assess this broadly, we performed a bead-based multiplex cytokine analysis of tumor lysates from mice maintained on either HFD-Soy (n-6) or HFD-Fish (n-3) diets. Across two independent experimental groups, several cytokines, including the IL-6 family members IL-6 and IL-11, trended lower in tumors from mice fed HFD-Fish (n-3) diet when compared to HFD-Soy (n-6) diet (Fig. 2*C*), although these differences did not reach statistical significance. We prioritized IL-6 family signaling for further investigation because of its established role in tumor–immune interactions and PDAC progression. In particular, oncogenic signaling can induce IL-6 early during tumorigenesis, establishing a feed-forward inflammatory circuit that promotes tumor initiation and progression (43). Together these findings identified IL-6 family signaling as a candidate pathway through which dietary PUFA composition may influence the inflammatory state of the PDAC TME.

### Dietary Omega-3 Fatty Acids Alter the PDAC TME Myeloid Cell Compartment.

To determine what cells were affected by different PUFA diets, we performed single-cell RNA sequencing on two sets of FACS-sorted CD45^+^CD3^+^ T cells, CD45^+^CD3^-^ non-T cell leukocytes, and CD45^-^YFP^+^ tumor cells. Each set of cells was isolated from two mice fed the HFD-Soy (n-6) diet and two mice fed the HFD-Fish (n-3) diet. Analysis of these datasets identified the expected immune populations, including multiple B and T lymphocyte subsets as well as large populations of neutrophils and myeloid cells (Fig. 2*D*). Among the major cell types, monocytes/macrophages had the largest number of differentially expressed genes between the HFD-Fish (n-3) and HFD-Soy (n-6) groups, indicating that this compartment was particularly responsive to diet (*SI Appendix*, Fig. S3*C*). Within this compartment, tumors from mice on the HFD-Fish (n-3) diet showed increased expression of antigen presentation and immune-interaction genes, including *H2-Ab1*, *H2-Eb1*, *H2-Aa,* and *Cd74*, together with *Cxcl16* and the complement-associated genes *C1qa*, *C1qb*, and *C1qc*, suggesting a shift towards a more immunologically engaged macrophage state with enhanced capacity for antigen handling and tissue surveillance. In contrast, monocytes/macrophages from tumors on the HFD-Soy (n-6) diet retained higher expression of genes associated with matrix remodeling, inflammatory tissue damage, and tumor-supportive TAM programs, including *Fn1*, *Vcan*, *Spp1*, *Arg1*, *Chil3*, *Lrg1*, and *Olr1* (Fig. 2*E*). This pattern is consistent with the hypothesis that dietary omega-3 fatty acids suppress a reparative, pro-tumor macrophage program while promoting a more antigen-presenting and less immunosuppressive myeloid state.

To further resolve the populations underlying these transcriptional differences, we subclustered the monocyte/macrophage compartment. This analysis identified one monocyte population and four distinct TAM populations (Fig. 2*F*). Tumors from mice on the HFD-Fish (n-3) diet showed a reduced proportion of *Arg1*^+^ TAMs and a corresponding increase in antigen-presenting TAMs (AP-TAMs) and *C1q*^+^ TAMs compared with tumors from mice on the HFD-Soy (n-6) diet (Fig. 2*F*). This decrease in the *Arg1*^+^ TAM subset was of particular interest because this subset is commonly associated with tumor-supportive and immunosuppressive macrophage programs (44–46) and suggested that dietary omega-3 fatty acids remodel macrophage states within the TME toward a less tumor-supportive state.

### TAM Subsets Have Distinct Spatial Localization in PDAC Tumors.

**Fig. 3.**
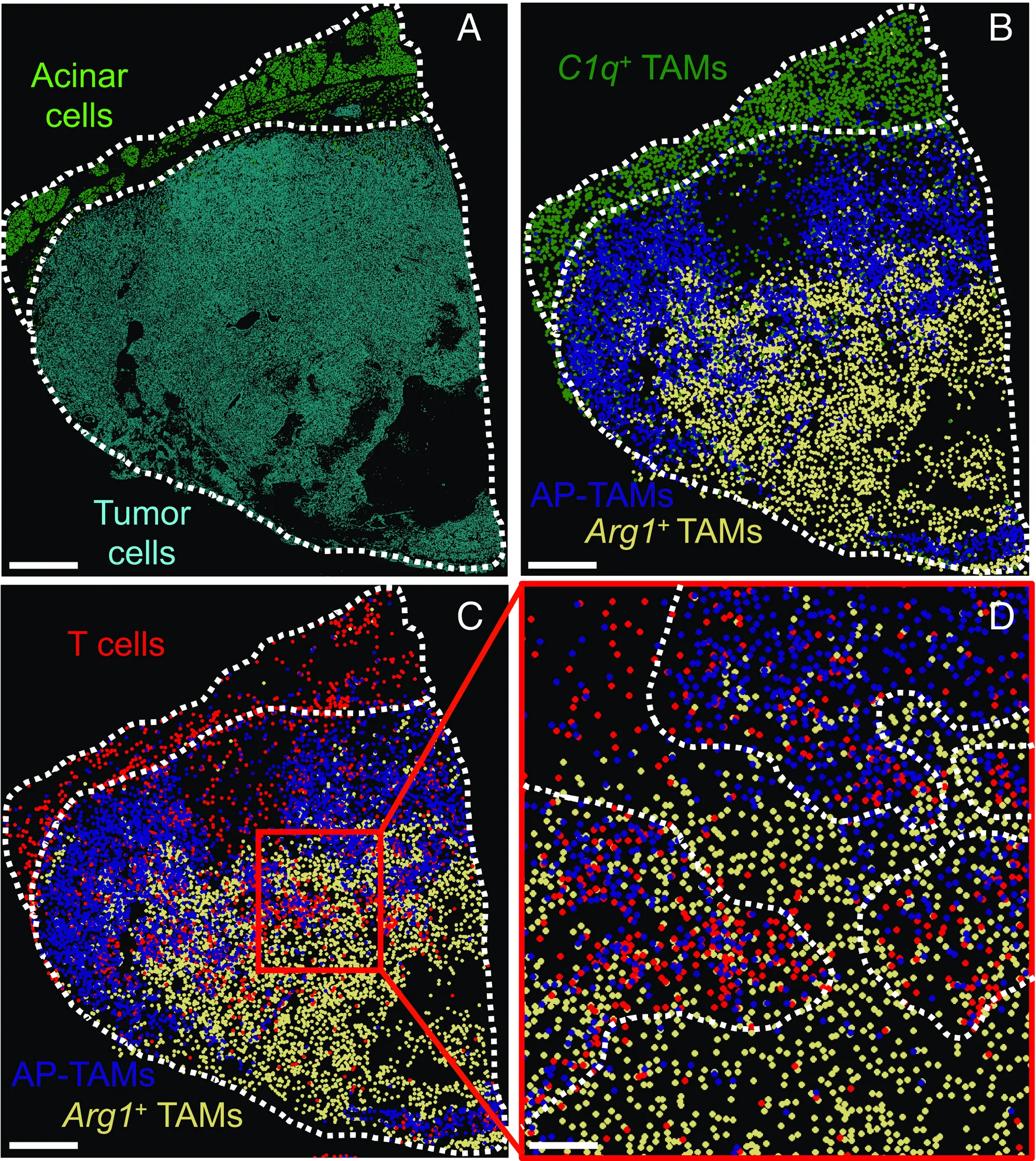
TAM subsets occupy distinct spatial niches within PDAC tumors. (*A*–*D*) Spatial transcriptomic analysis of orthotopic 6419c5 tumors maintained on HFD-Fish (n-3) diet using the 10X Xenium platform and a 480 gene panel. (*A*) Spatial distribution of normal acinar cells (green) and tumor cells (cyan). (*B*) Spatial distribution of overlays of *C1q*^+^ TAMs (green), *Arg1*^+^ TAMs (yellow), antigen-presenting TAMs (AP-TAMs, blue). (*C*) Spatial localization of overlays of AP-TAMs (blue) and *Arg1*^+^ TAMs (yellow) relative to T cells (red). (*D*) Higher magnification view of the box in (*C*). Dotted line encircles areas of closely associated AP-TAMs and T cells. Images from n = 1 biologically independent tumor. Spatial transcriptomic analysis was performed on tumors collected 21 days after orthotopic implantation. Data are representative of 1 independent experiment. [Scale bars, 1 mm (*A*–*C*) and 350 μm (*D*).]

To further understand how these macrophage subsets are organized within tumors, we next performed spatial transcriptomics. This analysis revealed that macrophage populations occupy distinct anatomical niches within the TME (Fig. 3 *A* and *B*). *C1q*^+^ TAMs were primarily localized at tumor margins and in the adjacent normal pancreas, whereas *Arg1*^+^ TAMs were enriched within necrotic and hypoxic tumor regions (Fig. 3*B* and *SI Appendix*, Fig. S4 *A* and *B*). Interestingly, the adjacent normal pancreas was enriched with *C1q*^+^ TAMs and where our previous MSI analysis found abundant omega-3 PUFAs (Fig. 1*E*), suggesting *C1q*^+^ TAMs are exposed to higher levels of omega-3 PUFAs whereas *Arg1*^+^ TAMs are exposed to lower levels. AP-TAMs were frequently observed in spatial clusters with CD4^+^ and CD8^+^ T cells, suggesting that they may participate in regulating local T cell activity, whereas *Arg1*^+^ TAMs were largely excluded from these immune niches (Fig. 3 *C* and *D*). Consistent with flow cytometry analyses (Fig. 2*A*), *Ly6C*^+^ monocytes were largely unchanged by diet and were primarily localized to the adjacent normal pancreas (*SI Appendix*, Fig. S4*C*), indicating that they likely play a limited role in the tumor-specific dietary effects observed here. Together, these findings indicate that TAM populations are both phenotypically and spatially heterogeneous within PDAC tumors. Combined with the MSI data this data supports that omega-3 lipids are enriched in regions with high concentrations of *C1q*^+^ TAMs and immune-interactive niches, linking lipid availability to macrophage state and function spatial within the TME.

### Omega-3 Fatty Acids Suppress TAM IL-6 Production, but Gp130 Signaling in Tumors Is Dispensable for Reduced PDAC Growth.

**Fig. 4.**
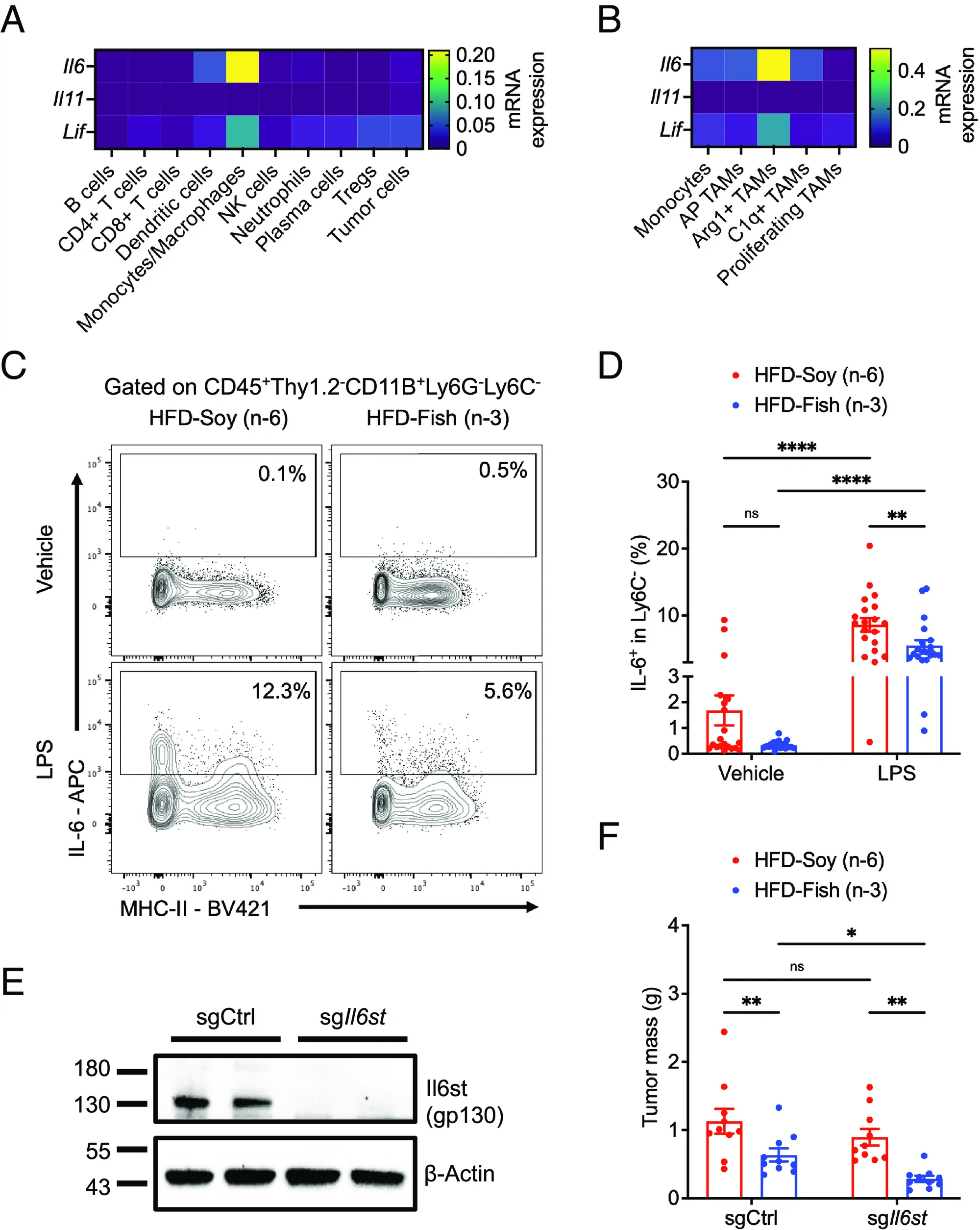
Dietary omega-3 fatty acids suppress macrophage IL-6 production independently of tumor-intrinsic gp130 signaling. (*A*) Expression of *Il6, Il11*, and *Lif* mRNA across cell populations in single-cell RNA sequencing (scRNA-seq) from Fig. 2*D*. (*B*) Expression of *Il6, Il11*, and *Lif* mRNA across monocyte/macrophage populations identified by scRNA-seq in Fig. 2*F*. (*C*) Representative flow cytometry plots of IL-6 production in tumor-infiltrating myeloid cells (CD45^+^Thy1.2^−^CD11b^+^Ly6G^−^Ly6C^−^) following ex vivo stimulation with LPS isolated from mice maintained on the indicated diets. (*D*) Quantification of IL-6^+^ myeloid cells (CD45^+^Thy1.2^−^CD11b^+^Ly6G^−^Ly6C^−^) with and without LPS stimulation from tumors shown in (*C*). (*E*) Western blot analysis of 6419c5 cells treated with single guide RNA targeting *Il6st* (sg*Il6st*) or a scramble control sequence (sgCtrl). (*F*) Endpoint tumor mass of orthotopic sg*Il6st* and sgCtrl 6419c5 tumors from mice maintained on the indicated diets. Representative data are shown from one (*A* and *B*) or three (*C*–*F*) independent experiments. Data are presented as mean ± SEM. Each dot represents one biologically independent mouse (*D* and *F*). Statistical analysis was performed using two-way ANOVA with Šídák’s multiple comparisons test (*D* and *F*). ns, not significant, *P < 0.05, **P < 0.01, ***P < 0.001, ****P < 0.0001.

The above findings suggested that dietary PUFA composition reshapes macrophage states within the TME and raised the possibility that altered cytokine production from these cells contributes to the observed tumor phenotype. Because earlier analyses showed reduced levels of IL-6-family cytokines in tumors from mice on the HFD-Fish (n-3) diet, we next investigated the cellular origin of these cytokines and whether their signaling directly mediated tumor suppression. Examination of the single-cell datasets revealed that IL-6 expression was restricted to the macrophage/monocyte cluster, whereas *Il11* expression was not detected at appreciable levels (Fig. 4*A*). Among the macrophage/monocyte populations, *Arg1*^+^ TAMs emerged as the dominant source of both *Il6* and *Lif* (Fig. 4*B*), indicating that this macrophage subset could account for the IL-6 family cytokine differences observed between dietary conditions. To determine whether dietary fatty acids altered the capacity of tumor-infiltrating myeloid cells to produce IL-6, we stimulated tumor-isolated single cell suspensions with the TLR4 agonist lipopolysaccharide (LPS) and analyzed intracellular IL-6 by flow cytometry. We found that most IL-6-producing myeloid cells were CD45^+^, CD11B^+^, and Ly6G^–^ (*SI Appendix*, Fig. S5*A*), consistent with our single-cell RNA-sequencing data associating *Il6* expression primarily with the monocyte/macrophage compartment (Fig. 4*A*). LPS treatment robustly induced IL-6 production within the TAM-enriched CD11B^+^Ly6G^–^Ly6C^–^ cells and this induction was ameliorated in the mice fed the HFD-Fish (n-3) diet when compared to mice fed the HFD-Soy (n-6) diet (Fig. 4 *C* and *D*). In the absence of LPS, IL-6 production showed a similar downward trend in the HFD-Fish group, but the difference was not statistically significant (Fig. 4*D*). These results suggested that dietary omega-3 fatty acids suppress the potential to produce inflammatory cytokines in TAMs.

Because IL-6 family cytokines signal through receptor complexes that share the common β-chain gp130 (*Il6st*), we tested whether tumor-intrinsic gp130 signaling contributed to diet-dependent differences in tumor growth. Genetic deletion of *Il6st* in 6419c5 tumor cells was confirmed by loss of gp130 protein (Fig. 4*E*) and found to reduce tumor growth in both dietary conditions. That is, *Il6st*-deleted tumors were approximately half the mass of controls in both HFD-Fish (n-3) and HFD-Soy (n-6) cohorts. However, tumors from the HFD-Fish (n-3) group remained smaller than those from the HFD-Soy (n-6) group even after *Il6st* deletion (Fig. 4*F*). These findings indicate that IL-6 family/gp130 signaling contributes to PDAC growth, but does not fully account for the differential tumor growth observed between the two diets, despite elevated IL-6 and IL-11 levels in the HFD-Soy (n-6) condition. Together, these results suggest that additional mechanisms beyond tumor-intrinsic gp130 signaling mediate the tumor-suppressive effects of the HFD-Fish (n-3) diet.

### Conversion of Omega-6 to Omega-3 Fatty Acids in Csf1r^+^ Myeloid Cells Reduces PDAC Tumor Size.

**Fig. 5.**
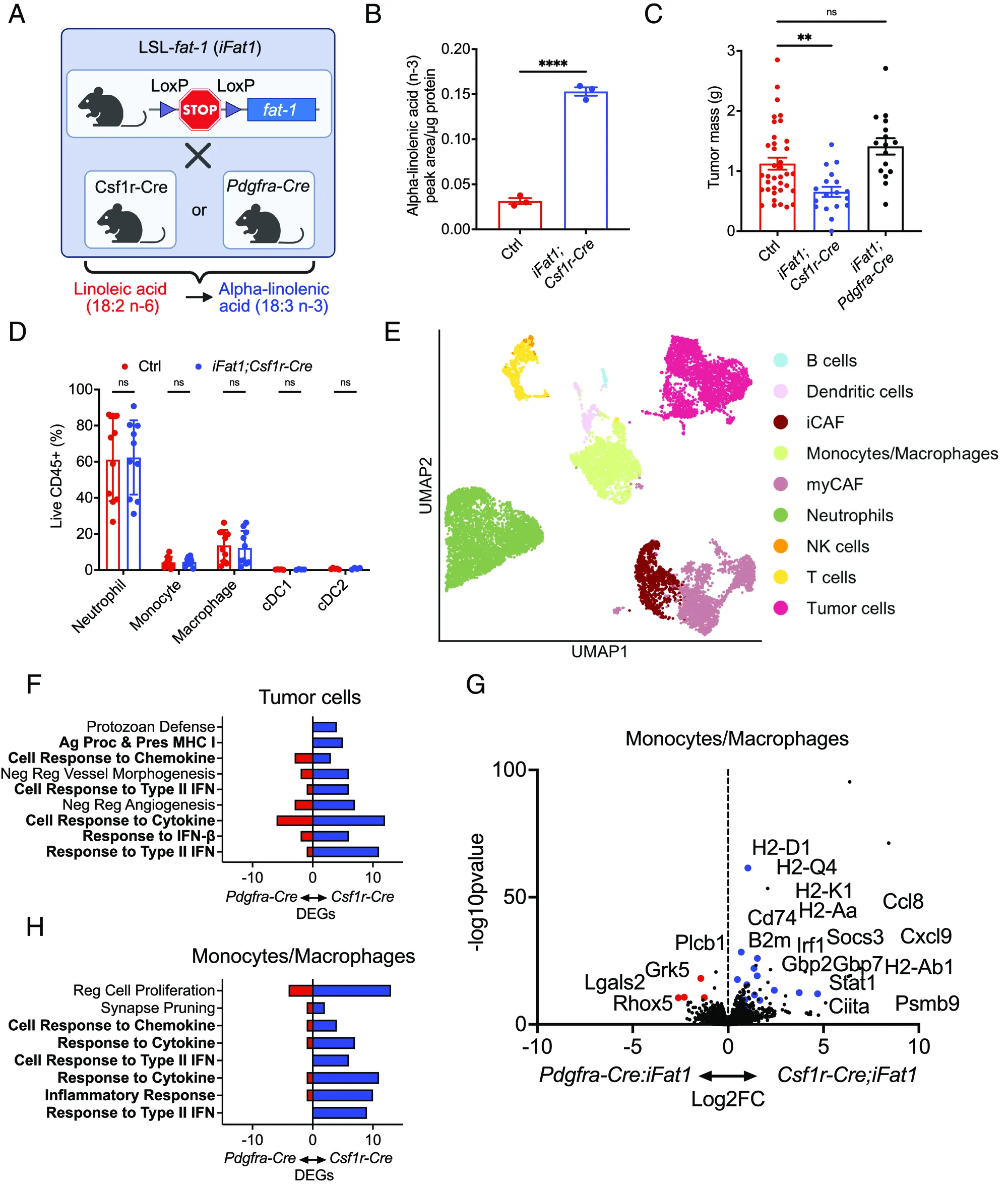
Myeloid-specific conversion of omega-6 to omega-3 fatty acids is sufficient to suppress PDAC tumor growth. (*A*) Experimental schematic of inducible *fat-1* (*iFat1*) expression in *Csf1r-Cre* or *Pdgfra-Cre* mice. (*B*) Mass spectrometric analysis of α-linolenic acid (ALA) in bone marrow–derived macrophages from control or *Csf1r-Cre;iFat1* mice following incubation with linoleic acid (LA). (*C*) Endpoint tumor mass of orthotopic 6419c5 tumors from control, *Csf1r-Cre*;*iFat1*, or *Pdgfra-Cre*;*iFat1* mice maintained on HFD-Soy (n-6) diet. (*D*) Flow cytometric quantification of major immune cell populations in orthotopic tumors from control or *Csf1r-Cre*;*iFat1* mice. (*E*) Annotated UMAP visualization of scRNA-seq data from sorted CD45^+^ leukocytes, CD45^–^YFP^+^ tumor cells, and CD45^–^YFP^–^Podoplanin^+^ fibroblasts taken from orthotopic tumors of *Csf1r-Cre*;*iFat1* or *Pdgfra-Cre*;*iFat1* mice maintained on HFD-Soy (n-6) diet. (*F*) Differential pathway enrichment analysis of tumor cells identified by scRNA-seq in *E*. (*G*) Volcano plot of differentially expressed genes in the monocyte/macrophage population identified in E comparing the indicated genotypes and highlighting type II interferon-response, inflammatory, and antigen-presentation genes. (*H*) Differential pathway enrichment analysis of macrophage/monocyte populations identified by scRNA-seq in E. Representative data from two (*B*, *D*, and *E*), four (*C*), or one (*F*–*H*) independent experiments. For scRNA-seq experiments, two tumors were pooled per sample with n = 2 samples per condition. Data are presented as mean ± SD (*B*) or ± SEM (*C* and *D*). Each dot represents one biologically independent mouse. Statistical analysis was performed using Welch’s *t* test (*B*), one-way ANOVA with Dunnett’s multiple comparisons test (*C*), using two-way ANOVA with Šídák’s multiple comparisons test (*D*), and differential expression analysis by Wilcoxon rank-sum test for scRNA-seq datasets. Ns, not significant, *P < 0.05, **P < 0.01, ***P < 0.001, ****P < 0.0001.

To determine whether the tumor-suppressive effects of omega-3 fatty acids arise from PUFA metabolism across multiple cell types or from cell-type-specific lipid metabolism, we asked whether local conversion of omega-6 to omega-3 fatty acids within defined cell types is sufficient to recapitulate the antitumor phenotype. To address this question, we utilized mice carrying an inducible version of the *fat-1* transgene (*iFat1*) from the nematode *Caenorhabditis elegans* (47), which encodes a desaturase capable of converting omega-6 PUFAs into omega-3 PUFAs by introducing a double bond at the omega-3 position (48). We focused on myeloid cells and fibroblasts given their abundance in the TME, their established roles in regulating PDAC growth (49), and the diet-associated changes observed in myeloid cells. To test whether myeloid cells could serve as a critical source of omega-3–mediated signaling, we generated mice in which *iFat1* expression was restricted to myeloid cells using a *Csf1r-Cre* driver (Fig. 5*A*). Validation experiments using bone marrow–derived macrophages confirmed that these cells were capable of converting the omega-6 fatty acid linoleic acid into the omega-3 fatty acid α-linolenic acid, as detected by mass spectrometry (Fig. 5*B*). When tested in orthotopic PDAC tumor models, mice with myeloid-specific *iFat1* expression fed the HFD-Soy (n-6) diet exhibited a 50% reduction in tumor mass (Fig. 5*C*). Although the two conditions were not directly compared within the *iFat1* cohort, the magnitude of this reduction was comparable to that observed in the separate HFD-Fish dietary experiment shown in Fig. 1*F*. In contrast, restricting *iFat1* expression to fibroblasts using a *Pdgfra-Cre* driver did not produce a similar reduction in tumor growth, suggesting that omega-3 signaling within the TME is highly dependent on the cellular context in which these lipids are generated (Fig. 5*C*). Notably, no differences in body mass were observed between *Csf1r-Cre*;*iFat1*, *Pdgfra-Cre*;*iFat1*, and control mice, supporting the previous evidence that the tumor-suppressive effects are not attributable to reduced weight gain (*SI Appendix*, Fig. S5*B*). Despite the reduction in tumor burden observed in mice with myeloid-specific *iFat1* expression, flow cytometric analysis of the TME did not reveal significant differences in the abundance of major immune cell populations compared with control tumors (Fig. 5*D*). Similarly, examination of macrophage subsets using markers such as CD206 and CD163 did not detect major shifts in macrophage composition (*SI Appendix*, Fig. S5*C*). Together, these studies establish macrophages as a critical metabolic determinant of PDAC tumor progression, demonstrating that their localized omega-3 fatty acid production is sufficient to suppress tumor progression.

To further investigate how myeloid-specific omega-3 PUFA production suppresses tumor growth and reshapes the TME, we performed single-cell RNA sequencing on tumors from mice conditionally expressing *iFat1* in either myeloid cells (*Csf1r-Cre*;*iFat1*) or fibroblasts (*Pdgfra-Cre*;*iFat1*), with both groups maintained on the HFD-Soy (n-6) diet throughout the experiment. Tumors were dissociated, and CD45^+^ leukocytes, YFP^+^ tumor cells, and Podoplanin^+^ fibroblasts were FACS-sorted for single-cell RNA sequencing (Fig. 5*E*). Comparison of tumor cells between the two conditions revealed enrichment of interferon signaling and antigen-presentation pathways in *Csf1r-Cre*;*iFat1* tumors relative to *Pdgfra-Cre;iFat1* tumors. These transcriptional differences were not accompanied by increased expression of apoptosis- or cell cycle–related genes that would otherwise explain the reduced tumor growth observed in *Csf1r-Cre*;*iFat1* mice (Fig. 5*F*).

We next examined whether immune cells exhibited similar transcriptional changes. Macrophages and monocytes from *Csf1r-Cre*;*iFat1* tumors showed enrichment of type II interferon-response, inflammatory, and antigen-presentation pathways compared with those from *Pdgfra-Cre;iFat1* tumors (Fig. 5 *G* and *H*). Among the genes contributing to these pathways, *H2-Aa*, *H2-Ab1*, and *Cd74* were upregulated in macrophages/monocytes from *Csf1r-Cre*;*iFat1* mice and mice fed the HFD-Fish (n-3) diet (Fig. 2*E*), identifying a conserved IFN-γ-responsive antigen-presentation program shared between the dietary and genetic models. Importantly, the pathway enrichment observed in Fig. 5*H* extends beyond these shared MHC class II genes, indicating broader activation of IFN-γ-responsive transcriptional programs in the myeloid compartment. Neutrophils from *Csf1r-Cre*;*iFat1* tumors similarly exhibited enrichment of innate immune sensing, lipid response, and extracellular matrix remodeling pathways, consistent with altered inflammatory and tissue-remodeling activity within the TME (*SI Appendix*, Fig. S5*D*). Together, these findings indicate that myeloid-specific omega-3 production induces a coordinated type II interferon-responsive program across both tumor and immune compartments, promoting antigen-presentation pathways without evidence of direct effects on tumor cell proliferation or survival.

## Discussion

Dietary lipid composition is increasingly recognized as a regulator of inflammation and tissue homeostasis, yet its role in shaping tumor–immune interactions in PDAC has remained poorly defined. Here, we show that shifting dietary PUFAs from omega-6 to omega-3 fatty acids remodels the PDAC TME, suppresses tumor growth, and reprograms myeloid cell states in a manner that is independent of T cells and systemic metabolic changes. A central finding of this study is that dietary PUFAs are incorporated into both structural phospholipids and phosphatidylinositol pools that support intracellular signaling, generating a spatially heterogeneous lipid microenvironment that does not simply mirror systemic lipid availability. This supports a model in which local lipid composition actively shapes cellular behavior within the TME.

Consistent with prior studies using constitutive *fat-1* models demonstrating that endogenous omega-3 production suppresses tumor growth, our data extend these observations by showing that the cellular source of omega-3 signaling is critical. Myeloid-specific conversion of omega-6 to omega-3 fatty acids was sufficient to recapitulate tumor suppression, whereas fibroblast-specific conversion was not. These data indicate that omega-3–dependent effects are not uniformly distributed across cell types or that the local lipid environment is heterogeneous and that total lipid content is not a good reflection of what cells are exposed to in the TME. The latter point is supported by the MSI data showing a heterogeneous distribution of lipids.

These findings raise several important questions. Do specific omega-3–derived lipid mediators directly reprogram macrophage function, or do they act indirectly through changes in membrane composition and signaling thresholds? How do macrophage–tumor and macrophage–stromal interactions integrate these lipid signals to influence tumor progression? Can these pathways be therapeutically targeted, either through dietary intervention or pharmacologic modulation of lipid metabolism and signaling?

These findings support a model in which macrophages act as key mediators of dietary lipid signaling within tumors. However, PUFA-derived mediators, particularly SPMs, can be generated through trans-cellular biosynthetic pathways involving multiple cell types (22). Thus, while conditional expression of *iFat1* using *Csf1r-Cre* demonstrates that macrophages are sufficient sources of omega-3 PUFAs to suppress tumor growth, the underlying mechanism remains unclear. One possibility is that macrophage-intrinsic n-3 PUFA metabolism generates downstream bioactive lipids, such as SPMs, that mediate tumor suppression. Alternatively, macrophage-derived n-3 PUFAs may act more directly by signaling to other cell types within the TME, but it is also possible that other cells receive higher amounts of n-3 PUFAs produced by *iFat1*-expressing macrophages that causes them to exert more pronounced effects on tumor growth.

Mechanistically, n-3 PUFA–enriched conditions reduced IL-6 family cytokine production and shifted macrophage populations away from Arg1+ tumor-supportive states toward C1q+ and antigen-presenting subsets. Importantly, these changes were accompanied by coordinated activation of interferon signaling and antigen presentation pathways in both macrophages and tumor cells, suggesting a broader shift in the inflammatory state of the TME from IL-6–associated signaling toward interferon-associated programs. Interestingly, the increase in antigen presentation pathways observed in macrophages and tumor cells differs from reports that n-3 PUFAs suppress antigen presentation in conventional antigen-presenting cells (26, 50). This discrepancy may reflect the distinct biology of the PDAC TME, where omega-3 fatty acids reduce tumor-supportive Arg1+ macrophages and induce interferon-associated programs rather than directly enhancing adaptive immune activation. Although transcriptional data do not directly capture proliferation or cell death, enrichment of interferon and antigen presentation pathways is consistent with a less permissive tumor state and increased immune visibility. Notably, these effects occurred independently of T cells, indicating that macrophage-driven inflammatory signaling may directly influence tumor cell state. Although n-3 PUFAs did not reduce cell cycle pathways at the mRNA level, our previous work showed that macrophage-driven interferon signaling in the TME can induce tumor cell quiescence and suppress tumor growth (51).

Several features of our findings suggest that dietary omega-3 fatty acids actively and continuously reshape the PDAC TME during tumor progression, raising the possibility that dietary lipid modulation could be therapeutically leveraged to restrain established disease. First, the tumor-suppressive effect of omega-3 fatty acids was rapid in onset yet required sustained exposure, arguing that dietary lipids actively shape the TME throughout tumor progression rather than simply establishing a long-lived primed state before tumor initiation. This finding is particularly relevant given that post-treatment dietary intervention alone was sufficient to suppress tumor growth, supporting the idea that modulation of dietary lipid composition may retain therapeutic potential even after disease establishment. In addition, the effect was dose-responsive and independent of body weight, indicating that tumor control was not secondary to weight differences or systemic metabolic alterations, consistent with recent work demonstrating direct effects of dietary fat composition on antitumor immunity (52). Finally, the absence of tumor suppression in female mice suggests that the therapeutic response to dietary omega-3 fatty acids may depend on sex-specific hormonal or metabolic factors that influence lipid signaling within the TME.

This study also has several limitations. While we identify macrophages as key responders to dietary lipid changes and implicate IL-6 family cytokines and interferon signaling, the precise lipid mediators responsible remain undefined. Recent work has demonstrated that macrophages can sense lipid classes via olfactory receptors (53). Whether these receptors, SPMs, or changes in membrane lipid composition directly drive macrophage reprogramming is not resolved. The potential contribution of CAF-derived cytokines, including IL-6, also warrants further investigation in contexts where stromal signaling may dominate. Additionally, the possibility of trans-cellular lipid mediator synthesis complicates attribution of function to a single cell type. We also did not directly assess circulating PUFA levels in the *Csf1r-Cre*;*iFat1* and *Pdgfra-Cre*;*iFat1* cohorts. Thus, although the genetic design supports a cell-intrinsic effect of lineage-restricted omega-3 production, we cannot exclude the possibility that *iFat1* expression altered systemic lipid availability. Conversely, serum lipid measurements may not fully capture the relevant biology, because localized omega-6-to-omega-3 conversion within the targeted lineage could alter cellular function without producing a detectable change in bulk circulating PUFA levels. Future work will be required to distinguish systemic from cell-intrinsic lipid remodeling and to map the cellular circuits that generate and respond to these signals.

In summary, this work demonstrates that dietary omega-3 fatty acids suppress PDAC growth through cell type–specific signaling within the TME, with macrophages emerging as key mediators of this effect. By linking dietary lipid composition to macrophage state, cytokine signaling, and tumor progression, these findings provide mechanistic insight into how nutritional factors can influence cancer biology and suggest new avenues for therapeutic intervention.

## Materials and Methods

Mice were maintained under specific pathogen-free conditions at the Salk Institute and all animal studies were approved by the Institutional Animal Care and Use Committee. Orthotopic PDAC tumors were established by implantation of KPC-derived tumor cell lines into the pancreas of mice maintained on defined high-fat diets. Diets were matched for macronutrient composition and differed primarily in lipid source, with omega-6–enriched diets derived from soybean oil and omega-3–enriched diets derived from menhaden fish oil. Tumor growth was monitored by ultrasound and tumors were harvested for downstream analyses.

Lipid composition of diets, serum, TIF, and tumor tissues was analyzed by targeted lipidomics and mass spectrometry imaging. Immune populations were characterized by flow cytometry and fluorescence-activated cell sorting. Cytokine concentrations were measured using multiplex bead-based assays. Single-cell RNA sequencing was performed on sorted tumor, immune, and stromal cell populations using the 10x Genomics platform. Spatial transcriptomic analyses were performed using the Xenium platform with a custom PDAC-focused gene panel.

Conditional omega-3 fatty acid production was achieved using Cre-dependent inducible fat-1 (*iFat1*) mice crossed to *Csf1r-Cre* or *Pdgfra-Cre* strains. Gene expression, pathway enrichment, and cellular state analyses were performed using standard bioinformatic workflows. Statistical analyses were conducted using GraphPad Prism. Additional details regarding animal models, diets, lipidomics, imaging, flow cytometry, sequencing, spatial transcriptomics, and statistical analyses are provided in the *SI Appendix*.

## Supplementary Material

Appendix 01 (PDF)

## Data Availability

scRNA-seq and spatial transcriptomics data have been deposited in Gene Expression Omnibus (GEO) under the accession numbers GSE334159 (54), GSE334158 (55), and GSE334315 (56). All study data are included in the article and/or *SI Appendix*.
