# Supplementary material for: Omega-3 fatty acids reprogram tumor-associated macrophage cytokine signaling and suppress pancreatic cancer growth
Source: Proc Natl Acad Sci U S A. 2026 Oct 16;123(42):e2618288123. doi: 10.1073/pnas.2618288123 (PMC13684287; doi:10.1073/pnas.2618288123)
Supplement: Supplementary file 1 [file pnas.2618288123.sapp.pdf]

PNAS

Supporting Information for

Omega-3 fatty acids reprogram tumor-associated macrophage cytokine signaling and suppress pancreatic cancer growth

Stephen Sakuma[a], Steven Zhao[a], Andrew Hildreth[a], Gloria Liu[a], Valisha Shah[a], Ming Sun[a], Shirong Tan[a], Emily Jacobs[a], Michael A. LaPorta[a], Jesse Furguiele[a], Natalia Avina-Ochoa[a], Anna-Maria Globig[a], Victoria Tripple[a], ImmgenT Consortium*, Geraldine Maier[b], Satchidananda Panda[b], Michelle L. Reyzer[c], Jing X. Kang[d,e], David W.L. Ma[f], Matthew Rodeheffer[g,h], Saame Raza Shaikh[i], and Susan M. Kaech[a]

Susan M. Kaech
Email: sue.kaech@alleninstitute.org

This PDF file includes:

Supporting information text
Figures S1 to S5
Tables S1 to S2
SI References

Supporting Information Text

* ImmgenT Consortium Authors:

Aaron Liu[1], Alexander Chervonsky[2], Alexandra Cassano[2], Alia Welsh[3], Amir Ferry[11], Ananda Goldrath[11], Andrea Lebron-Figueroa[5], Ankit Malik[2], Anna-Maria Globig[4], Antoine Freuchet[2], Bana Jabri[2], Charlotte Imianowski[6], Christophe Benoist[5], Claire Thefaine[7], Dan Kaplan[6], Dania Mallah[5], Dario Vignali[6], David Sinclair[5], David Zemmour[2], Derek Bangs[8], Domenic Abbondanza[2], Enxhi Ferraj[9], Eric Weiss[6], Erin Lucas[7], Evelyn Chang[9], Gavyn Bee[10], Giovanni Galletti[11], Ian Magill[5], Iliyan D Iliev[12], Jinseok Park[5], Joonsoo Kang[9], Jordan Voisine[2], Josh Choi[5], Julia Merkenschlager[13], Jun R. Huh[5], Katharine Block[7], Ken Cadwell[10], Kennidy K. Takehara[11], Kevin Osum[7], Laurent Brossay[14], Laurent Gapin[15], Liang Yang[5], Lizzie Garcia-Rivera[1], Marc K. Jenkins[7], Maria Brbic[16], Maria-Luisa Alegre[2], Marion Pepper[8], Mariya London[17], Matthew Stephens[2], Maurizio Fiusco[16], Melanie Vacchio[3], Michael Starnbach[5], Michel Nussenzweig[13], Mitch Kronenberg[18], Myriam Croze[19], Nalat Siwapornchai[5], Nathan Morris[12], Nicole E. Scharping[11], Nika Abdollahi[19], Nitya Mehrotra[2], Odhran Casey[5], Olga Barreiro del Rio[5], Paul Thomas[20], Peter Carbonetto[2], Remy Bosselut[3], Rocky Lai[9], Sam Behar[9], Sam Borys[14], Sara E. Hamilton[7], Sara Mostafavi[8], Sara Quon[11], Serge Candéias[21], Shanelle Reilly[14], Shanshan Zhang[5], Siba Smarak Panigrahi[16], Sofia Kossida[19], Stefan Muljo[3], Stefan Schattgen[20], Stefani Spranger[22], Steve Jameson[7], Susan M. Kaech[1], Takato Kusakabe[12], Taylor Heim[22], Tianze Wang[8], Tomoyo Shinkawa[9], Ulrich von Andrian[5], Val Piekarsa[5], Véronique Giudicelli[19], Vijay Kuchroo[5], Woan-Yu Lin[12], Ziang Zhang[2]

* ImmgenT Consortium Affiliations:

1. NOMIS Center, Salk Institute for Biological Sciences, 2. The University of Chicago, 3. National Institutes of Health, 4. Allen Institute for Immunology, 5. Harvard Medical School, 6. Dept of Dermatology and Immunology, University of Pittsburgh, 7. University of Minnesota, 8. University of Washington, 9. UMass Chan Medical School, 10. University of Pennsylvania, 11. University of California San Diego, 12. Weill Cornell Medicine, 13. The Rockefeller University, 14. Brown University, 15. University of Colorado Anschutz Medical Campus, 16. Swiss Federal Institute of Technology, Lausanne, 17. New York University, 18. La Jolla Institute, 19. IMGT, Université de Montpellier, 20. St. Jude Children's Research

Hospital, 21. Alternative Energies and Atomic Energy Commission, Grenoble, 22. Massachusetts Institute of Technology

Extended Experimental Methods

Mice

Mice were housed under specific pathogen-free conditions at the Salk Institute. All studies were conducted in accordance with institutional IACUC protocols. Male mice were used unless otherwise noted. C57BL/6J mice (Strain #:000664), Csf1r-Cre mice (Strain#: 029206), and Pdgfra-Cre mice (Strain #:013148) were purchased from Jackson Laboratories. Cre-inducible *fat-1* (*iFat1*) mice were a gift from Dr. David Ma. Mice were bred in-house to generate *iFat1*; *Csf1r-Cre* and iFat1; Pdgfra-Cre mice. Mice were maintained on standard mouse diet until they were 6 weeks of age before dietary manipulation. Standard mouse diet was PicoLab Rodent Diet 20 (cat. 5053). High Fat diets were obtained from Research Diets and had identical in macronutrient composition except for fat source; Fish (cat. D03022403i), Soy (cat. D05042003i), or 50% Fish/50% Soy (cat. D22011715i). Fish source was menhaden and soy source was soybeans. Blood was collected by terminal cardiac puncture at the experimental endpoint and serum was isolated by centrifugation. Serum amylase and lipase levels were measured by IDEXX BioAnalytics using standard clinical chemistry assays according to the manufacturer's protocols. Food intake and oxygen consumption were simultaneously measured for individually housed mice with a LabMaster system (TSE Systems). Mice were acclimatized for 3 days before 5 days of data collection.

Cell culture

6419c5 and 2838c3 cells were obtained from Kerafast and FC1199 cells were donated by the Engle lab. 6419c5 and 2838c3 cells were maintained in cultured in DMEM/F12 medium (Gibco) with 10% FBS, 1% penicillin/streptomycin, and 1% Minimum Essential Medium non-essential amino acids. Cells were used for fewer than 10 passages and regularly tested for Mycoplasma. For tumor engraftment, cells were dissociated using 0.25% Trypsin-EDTA(Gibco), resuspended to single-cell suspensions in PBS. CRISPR RNP was used to generate *Il6st* KO cell lines. Briefly, single guide RNA (sgRNA) targeting *Il6st* gene or a scramble control sequence was incubated with Cas9 nuclease (IDT, catalog #1081059) to generate the RNP complex and then delivered to cell lines by electroporation

using Lonza SF Cell Line 4D-Nucleofector X Kit (catalog #V4XC-2032, program A549). After electroporation, cells were cultured in fresh medium for 3 days and then single cell cloned to identify clones with homogeneous knockout.

Pancreatic tumors

Mice were anaesthetized under continuous isoflurane and given 1 mg/kg buprenorphine slow release. The left abdominal side was shaved to expose the incision site, which was sterilized by two rounds of povidone–iodine and isopropanol. A small incision in the abdominal skin was made to expose the underlying muscle. A second small incision was made in the muscle layer, and the pancreas was gently exposed from the abdominal cavity. A 10 ul mixture of 50% Matrigel (Corning 356231) and 50% DMEM with 500 cells was injected using a 50 ul Hamilton syringe with a 26-gauge needle (BD) into the tail of the pancreas. The pancreas was returned into the abdominal cavity, and the incision site was closed using sutures (Covidien, UL213) for the inner muscle layer and 9 mm steel skin clips for the outer skin. All surgical tools were sterilized between animals using 70% isopropyl alcohol and a heated bead sterilizer. Animals were monitored for 3 days, and skin clips were removed 10 days after surgery. For growth monitoring of PDAC, mice were subjected to abdominal ultrasound at day 11, 14, and 18 after implantation using a Vevo 3100 ultrasound machine (Visualsonics). For T cell depletion, mice were treated with anti-mouse CD4 (clone GK1.5) and anti-mouse CD8α (clone 2.43) antibodies or isotype control IgG2A (clone 1-1) (Leinco) at 200 µg per mouse per injection. Antibodies were administered twice during the week prior to tumor implantation and then once weekly thereafter to maintain depletion throughout the experiment. Unless otherwise noted were conditioned on high-fat diet for six weeks before tumors were implanted orthotopically. Tumors and associated tissues were collected for immunologic and histologic analysis at day 19 except for 2838c3 tumors which were collected at day 38.

Lipidomics

Targeted lipid analysis was performed at the LIPID MAPS Lipidomics Core at UC San Diego[1,2]. Serum, tumor interstitial fluid (TIF), and tumor tissue samples were subjected to targeted lipidomic analysis to quantify free fatty acids (FFAs), total fatty acids, and phospholipid species, including phosphatidylcholine (PC) and phosphatidylinositol (PI). Lipids were extracted using organic solvent-based methods adapted from established Folch and acidified extraction protocols to enable recovery of both neutral and polar lipid

species, with internal standards added prior to extraction for normalization and relative quantification. Free (non-esterified) fatty acids were isolated, derivatized, and analyzed by gas chromatography–mass spectrometry (GC/MS, Agilent) using a stable isotope dilution approach to quantify individual omega-3 and omega-6 species. For total fatty acid analysis, lipid extracts were hydrolyzed to release esterified fatty acids prior to GC/MS quantification. Phospholipid species were analyzed by liquid chromatography–mass spectrometry (LC/MS) following reverse-phase chromatographic separation using a Vanquish UHPLC (Thermo) and detection by electrospray ionization tandem mass spectrometry in TopN monitoring mode using a Q-Exactive Orbitrap Mass spectrometer (Thermo), with lipid identification based on precursor and fragment ions using Lipid Data Analyzer software and quantification performed relative to class-specific internal standards. Samples were diluted as necessary to maintain measurements within the linear dynamic range. All lipid species were quantified as relative abundance normalized to internal standards, enabling comparison of omega-3 and omega-6 incorporation across free fatty acid pools and membrane phospholipid classes.

Flow cytometry and cell sorting

Tumors were harvested at defined experimental endpoints and mechanically minced. Minced tumors were processed in dissociation buffer containing collagenase IV, Dispase II, and DNase I in RPMI supplemented with 1% penicillin–streptomycin, HEPES, and sodium pyruvate, and incubated at 37 °C for 30 minutes with agitation. Following digestion, samples were passed through a 70 µm cell strainer, washed, and resuspended in RPMI containing 10% fetal bovine serum (FBS). Red blood cell lysis was performed where necessary using ACK lysis buffer. Cells were counted and prepared for staining. For flow cytometric analysis, cells were first incubated with a fixable viability dye in PBS to exclude dead cells, followed by staining with fluorophore-conjugated antibodies against surface markers in PBS supplemented with 1% FBS for 20–30 minutes on ice. For intracellular cytokine detection, cells were stimulated with 1 µg/ml LPS in the presence of 5 µg/ml brefeldin A at 37 °C for 5 hours. Cells were then fixed with Fixation Buffer (Biolegend cat. 420801) and permeabilized using Fixation/Permeabilization Concentrate (eBioscience cat. 00-5123-43) diluted in Fixation/Permeabilization Diluent (eBioscience cat. 00-5223). Permeabilized cells were stained with antibodies against intracellular targets. Data acquisition was performed on a BD A3 or LSRII flow cytometer, and data were analyzed using FlowJo software. For cell sorting applications, tumor suspensions were surface

stained as described above and sorted using a BD FACSAria cell sorter under sterile, temperature-controlled conditions. The following list of viability stains was used for flow cytometry or cell sorting: Live/Dead Ghost e780 (1:1000; Tonbo Biosciences, cat. #13-0875-T100), Live/Dead Zombie Aqua (1:1000; Biolegend, cat. #423101). The following list of antibodies and concentrations was used for flow cytometry or cell sorting: anti-TruStain FcX (CD16/32) (1:50; BioLegend, cat. #101320, RRID: AB_312801), anti-Ly6G (1:200; BD Biosciences, cat. #563978, RRID: AB_2738520), anti-CD11b (Mac-1) (1:400; BD Biosciences, cat. #612800, RRID: AB_2738811), anti-I-A/I-E (MHC II) (1:500; BioLegend, cat. #107632, RRID: AB_2650896), anti-Ly6C (1:200; BioLegend, cat. #128036, RRID: AB_2562353), anti-CD45 (1:200; BioLegend, cat. #103147, RRID: AB_2564383), anti-F4/80 (1:200; BioLegend, cat. #123114, RRID: AB_293478), anti-iNos (1:200; BioLegend, cat. #696806, RRID: AB_2876745), anti-Arg1 (1:100; Thermo Fisher Scientific (eBioscience), cat. #46-3697-82, RRID: AB_2734843), anti-C1q (1:200; BD Biosciences, cat. #569965, RRID: AB_3685438), anti-IL-6 (1:100; BioLegend, cat. #504508, RRID: AB_10694868), anti-Podoplanin (1:200; BioLegend, cat. #127410, RRID: AB_10613649), anti-MHC-I (H-2Kd) (1:200; BioLegend, cat. #116608, RRID: AB_313743), anti-PD-L1 (CD274) (1:200; BioLegend, cat. #124314, RRID: AB_10643573), anti-CD206 (MMR) (1:100; BioLegend, cat. #141704, RRID: AB_10901166), anti-CD64 (1:200; BioLegend, cat. #139314, RRID: AB_2563904), anti-CD11c (1:100; BioLegend, cat. #117339, RRID: AB_2562414), anti-XCR1 (1:400; BioLegend, cat. #148204, RRID: AB_2563843), anti-CD163 (1:100; BioLegend, cat. #155315, RRID: AB_2890708), anti-Pro-IL-1β (1:100; Thermo Fisher Scientific (eBioscience), cat. #25-7114-82, RRID: AB_2573526), anti-IFNγ (1:100; BioLegend, cat. #505830, RRID: AB_2563105), anti-TNFα (1:100; BioLegend, cat. #506306, RRID: AB_315427), anti-FoxP3 (1:100; Thermo Fisher Scientific (eBioscience), cat. #11-5773-82, RRID: AB_465243), anti-CD4 (1:400; BioLegend, cat. #100536, RRID: AB_493701), anti-CD4 (1:400; BD Biosciences, cat. #612761, RRID: AB_2870092), anti-CD8a (1:400; BioLegend, cat. #100748, RRID: AB_2562100), anti-CD8a (1:400; BioLegend, cat. #100722, RRID: AB_312761), anti-Thy1.2 (CD90.2) (1:200; BioLegend, cat. #105328, RRID: AB_10613293), anti-PD-1 (CD279) (1:400; BioLegend, cat. #135225, RRID: AB_2563680), anti-CX3CR1 (1:200; BioLegend, cat. #149023, RRID: AB_2565706), anti-SlamF6 (CD352) (1:100; BD Biosciences, cat. #745250, RRID: AB_2742834), anti-Tim-3 (CD366) (1:200; BioLegend, cat. #119727, RRID: AB_2716208), anti-CD101 (1:200; Thermo Fisher Scientific (eBioscience), cat. #12-1011-82, RRID: AB_2572573), anti-Tox (1:100; Miltenyi Biotec, cat. #130-118-474, RRID: AB_2751522),

anti-CD44 (1:200; BioLegend, cat. #103028, RRID: AB_830785), anti-NK1.1 (1:400; BioLegend, cat. #108710, RRID: AB_313397), anti-CD3 (1:400; Thermo Fisher Scientific (eBioscience), cat. #17-0031-82, RRID: AB_469315), anti-CD19 (1:400; BioLegend, cat. #115512, RRID: AB_313643).

Cytokine array

Lysates were prepared from snap-frozen pancreatic tumors and maintained on ice throughout processing. Tumors were homogenized in ice-cold PBS supplemented with protease inhibitors (Roche cOmplete Mini, EDTA-free) using a bead mill or rotor–stator homogenizer while minimizing heat generation. Lysates were clarified by 2 times centrifugation at ≥16,000 × g for 10 min at 4 °C, to remove particulate matter. Protein concentration was measured using a Bradford assay (Bio-Rad, cat. #161-0406) in a 96-well format. Concentrations were calculated from a standard curve within the linear range. Total protein concentrations were adjusted to 3.8 mg/mL, and samples were aliquoted into endotoxin-free tubes and shipped on dry ice. Cytokines were measured by Mouse Cytokine/Chemokine 68-Plex Discovery Assay Arrays (MD68) by Eve Technologies.

Mass spectrometry imaging

Matrix-assisted laser desorption/ionization mass spectrometry imaging (MALDI-MSI) was performed at the Vanderbilt Mass Spectrometry Research Center (MSRC). Fresh-frozen tumor tissues were sectioned at 12 µm thickness using a cryostat and mounted onto conductive indium tin oxide (ITO)-coated glass slides. Tissue sections were flushed with nitrogen gas, put in a sealed slide holder, and stored at -80°C until ready for analysis. Slides were removed from the freezer and allowed to warm up to room temperature for at least 30 minutes in a benchtop desiccator before opening the slide holder. Matrix application was performed using an automated sprayer to ensure uniform coverage and reproducible ionization across samples. 9-Aminoacridine (9AA) was made as a 5 mg/ml solution in 90% methanol and applied to the slides via a M3 TM Sprayer (HTX Technologies). The sprayer conditions were as follows: 4 passes in a criss-cross pattern, 0.15 ml/min flow rate, 700 mm/min stage speed, 2 mm track spacing, 10 psi, 70°C nozzle temperature. Negative ion mode mass spectra were acquired across the full tissue section in a rasterized manner at 10 µm pitch. Relative quantification of metabolites was performed by comparing ion intensities across conditions following normalization to total ion current. MSI datasets were processed using Bruker SCiLS lab. Ion images were

reconstructed and visualized to assess spatial distribution of lipid species across tumor and adjacent tissue regions. Annotation of lipid species was performed based on accurate mass matching using a mass tolerance of ±0.005 m/z (~10 ppm), with putative identifications assigned using the LipidMaps database. Representative ion images were generated for linoleic acid (LA; 18:2, n-6; m/z 279.232 $[M-H]^-$), arachidonic acid (AA; 20:4, n-6; m/z 303.231 $[M-H]^-$), eicosapentaenoic acid (EPA; 20:5, n-3; m/z 301.217 $[M-H]^-$), and docosahexaenoic acid (DHA; 22:6, n-3; m/z 327.231 $[M-H]^-$). To enable integration with histology datasets, MSI data were co-registered with histological images using ImageJ. This approach allowed correlation of lipid distributions with histological features.

Single-cell RNA sequencing (scRNA-seq)

Two PDAC tumors were pooled for each sample and each condition contained two samples. Cells were stained for sorting with a BD FACSAria as described above. Sorted cells were spun down at 420 r.c.f. at 4 °C for 4 min, resuspended in PBS with 2% FBS. Samples were pooled for encapsulation into gel bead-in-emulsions using a chromium controller (10X Genomics) with a targeted cell recovery of 10,000 cells. Libraries were generated following the manufacturer’s instructions of the Chromium Next GEM SingleCell 3′ Reagent Kit v.3.1 (10X Genomics) followed by sequencing on an Illumina NovaSeq6000 or Element Aviti. Sequencing was performed to a depth of approximately 20,000–40,000 reads per cell, using paired-end reads (28 bp for read 1 and 91 bp for read 2), achieving a sequencing saturation of approximately 30% across samples. Sequencing data were demultiplexed and aligned to the mouse reference genome (GRCm39, 2024-A) using the Cell Ranger Multi pipeline (v9.0). After which the count matrix was processed using Seurat v5.4.0. Downstream analysis was performed using Scanpy (v1.11.2), AnnData (v0.11.4), and scvi-tools (v1.4.2). Integrated analysis of RNA and protein modalities was conducted using the totalVI model within scvi-tools, followed by Leiden clustering on the joint latent space. Quality control filtering retained cells with total RNA counts within the 5th to 95th percentile. Cells with mitochondrial gene expression exceeding 1.5% were excluded. Doublets were identified and removed using Scrublet. Both the datasets were manually annotated using DEGs for leiden clusters. Differentially expressed genes between conditions were then identified within each annotated cell population using Wilcoxon rank-sum testing implemented via the rank_genes_groups function.

For diet comparison experiments (HFD-Soy (n-6) versus HFD-Fish (n-3)), cells were labelled with Biolegend TotalSeq-C hashtag antibodies found in Table S1 before sorting during surface staining. Sorted live CD45$^+$CD3$^+$ T cells, CD45$^+$CD3$^-$ non-T cell leukocytes, and CD45$^-$YFP$^+$ tumor cells were combined prior to encapsulation at proportions of 40%, 50%, and 10%, respectively. Raw gene expression and antibody-derived tag matrices were then processed using the Python packages Scanpy and Muon. Cells were additionally filtered based on total protein counts, retaining those with counts between 100 and 20,000. Sample multiplexing hashtags were used for demultiplexing with hashsolo, and hashtag doublets and negative droplets were discarded.

For iFat1 experiments, Sorted live CD45$^+$ leukocytes, CD45$^-$YFP$^+$ tumor cells, and CD45$^-$YFP$^-$Podoplanin$^+$ fibroblasts were combined prior to encapsulation at proportions of 60%, 20%, and 20%, respectively. A custom reference panel was built adding the Cre[3], YFP[4], and iFat1[5] gene sequences.

Spatial transcriptomics

Spatial transcriptomics was performed as previously described[6] using the 10X Xenium platform. Tumors were collected 21 days after tumor implantation from mice on HFD-Fish (n-3) diet. FFPE tissues were sectioned at 5 µm thickness onto Xenium slides, followed by deparaffinization and permeabilization according to manufacturer protocols (10x Genomics user guides CG000578 and CG000580). Probe hybridization, ligation, and rolling circle amplification were performed following the Xenium workflow (CG000582). Briefly, probes were hybridized overnight at 50 °C at a concentration of 10 nM using a custom panel targeting 480 transcripts. Gene panel design was based on prior single-cell RNA sequencing datasets and curated marker genes representing tumor, immune, and stromal compartments, supplemented with ligand–receptor interactions within PDAC tumors (Table S2). After stringent washing, probes were ligated at 37 °C for 2 h, followed by enzymatic amplification to generate barcode signals corresponding to individual RNA molecules. Background fluorescence was quenched prior to imaging, and slides were loaded into the Xenium Analyzer for high-resolution imaging. Raw imaging data were processed using the Xenium Ranger pipeline for transcript decoding, cell segmentation, and assignment of transcripts to individual cells. Cells were excluded if they were more than 5 median absolute deviations from the median in log-transformed total counts, if they had more than 1,500 total transcripts, a cell area greater than 500 μm^2, or fewer than 3

detected genes. Downstream analysis was performed using Scanpy and scvi-tools, including dimensionality reduction with scVI (n_latent = 30) and Leiden clustering (resolution = 1.0). Cell types were assigned using cluster-specific differential gene expression and canonical marker genes. Raw images were generated in the 10x Genomics Xenium Explorer 4.1.1. Image overlays were generated using ImageJ. Multichannel images were split into RGB channels, local intensity maxima in each channel were identified using distance mapping and peak detection. Images were rendered as color-coded dots on a composite image to visualize relative spatial distributions of cell types.

Western blotting

To make protein extracts, cells were lysed with RIPA buffer containing 1x protease inhibitors (Pierce TM Protease Inhibitor Mini Tablets, EDTA-free, Sigma) and phosphatase inhibitors (PhosSTOP, Roche). Homogenates were sonicated for 10 seconds, incubated on ice for 5 minutes, and spun down for 10 minutes. Laemmli Sample Buffer (Bio-rad) premixed with β-mercaptoethanol was added to samples to 1X concentration before incubation for 5 minutes at 90°C. For western blot analysis, lysates extracted from 2x10^5 cells were resolved by SDS-PAGE on Mini-PROTEIN TGX Precast Protein Gels (Bio-rad) and transferred to PVDF Membranes (Bio-rad). Membranes were blocked for 1 hour at RT in blocking buffer composed of TBS-0.1% Tween 20 (TBS-T) containing 5% BSA and incubated with primary antibodies overnight at 4°C in blocking buffer. After three washes with TBS-T, the secondary antibody was added and incubated for 1 hour at RT in blocking buffer. Membranes were then washed with TBS-T for three times and developed using SuperSignal West Pico PLUS Chemiluminescent Substrate (ThermoFisher Scientific). Signal was acquired with a Bio-Rad Chemidoc XRS+ molecular imager using the ImageLab software. The following list of antibodies and concentrations were used for western blotting: anti-GP130/CD130 (1:2000; Thermo Fisher Scientific, cat. #A304-292A, RRID: AB_2621123), anti-β-Actin (1:2000; Cell Signaling Technology, cat. #8457, RRID: AB_10950489), goat anti-rabbit IgG (H+L) HRP (1:2000; Thermo Fisher Scientific, cat. #31460, RRID: AB_228341).

Immunohistochemistry

Tumor tissues were fixed in 10% neutral buffered formalin (LabChem, LC146702) for at least 18 hours. Fixed tissues were stored in 70% ethanol and submitted to the Tissue

Technology Shared Resource Core at the University of California San Diego Moores Cancer Center for paraffin embedding, sectioning and immunohistochemistry (IHC). For IHC, tissue was baked at 60 °C before clearing and rehydrating through successive alcohols (3× xylene,2× 100% ethanol, 2× 95% ethanol, 2× 70% ethanol, diH2O). Hematoxylin and eosin staining was used to assess morphology, and Mason's Trichrome staining was used to assess fibrosis.

Software and Statistics

Experimental schematics were created with Biorender.com. Tumor volumes were measured by ultrasound and analyzed using standard radiologic quantification software. FlowJo (v.10.10.1) was used for analysis of cytometric data. Original data plots are depicted using FlowJo as 2% contour plot with outlier setting. CalR[7] Version 2 was used for food intake and oxygen consumption analysis. Statistical analysis was performed using GraphPad (v.11 Prism Software). Statistical tests used are indicated in the figure legends. Comparisons between two groups used unpaired two-tailed t-tests or Mann–Whitney tests when applicable. Multiple-group comparisons used one- or two-way ANOVA with correction for multiple testing. $P < 0.05$ was considered significant, whereby ****$P < 0.0001$, ***$P < 0.001$, **$P < 0.01$ and *$P < 0.05$. All experiments were independently replicated unless otherwise noted, and sample sizes were informed by prior orthotopic PDAC studies and power calculations were performed.

Figures

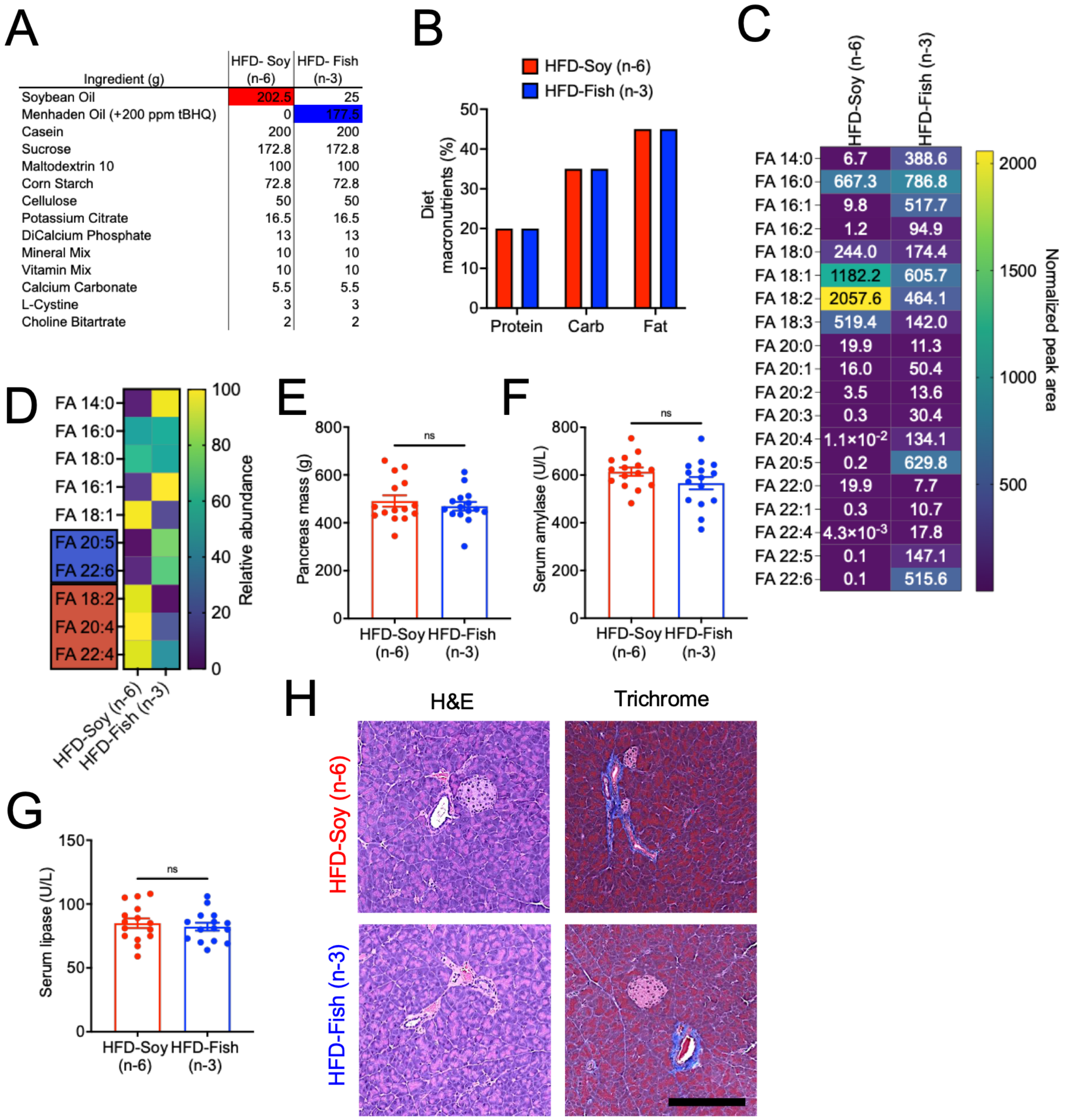

Fig. S1. Characterization of omega-3 and omega-6 dietary interventions and effects on baseline pancreatic tissue. (A) A table showing the ingredient composition of the diets. **(B)** Ingredient extrapolated macronutrient diet composition. **(C)** Lipidomic analysis of diet total fatty acid species in the indicated diets. **(D)** Lipidomic analysis of serum free fatty acid species from mice maintained on the indicated diets for 6 weeks. **(E)** Pancreas mass from mice maintained on the indicated diets in the absence of tumor. **(F)** Serum amylase levels from mice maintained on the indicated diets. **(G)** Serum lipase levels from mice maintained on the indicated diets. **(H)** Representative hematoxylin and eosin (H&E) and Masson's trichrome staining of pancreatic tissue from mice maintained on the indicated diets. Representative histological images are shown from n = 8 (HFD-Soy (n-6)) or 9 (HFD-Fish (n-3)) biologically independent mice per group. Scale bar, 600 µm. Representative data are shown from 2 (C-H) independent experiments. Data are

presented as mean ± SEM. Each dot represents one biologically independent mouse. Statistical analysis was performed using Welch's t test (E-G). ns, not significant.

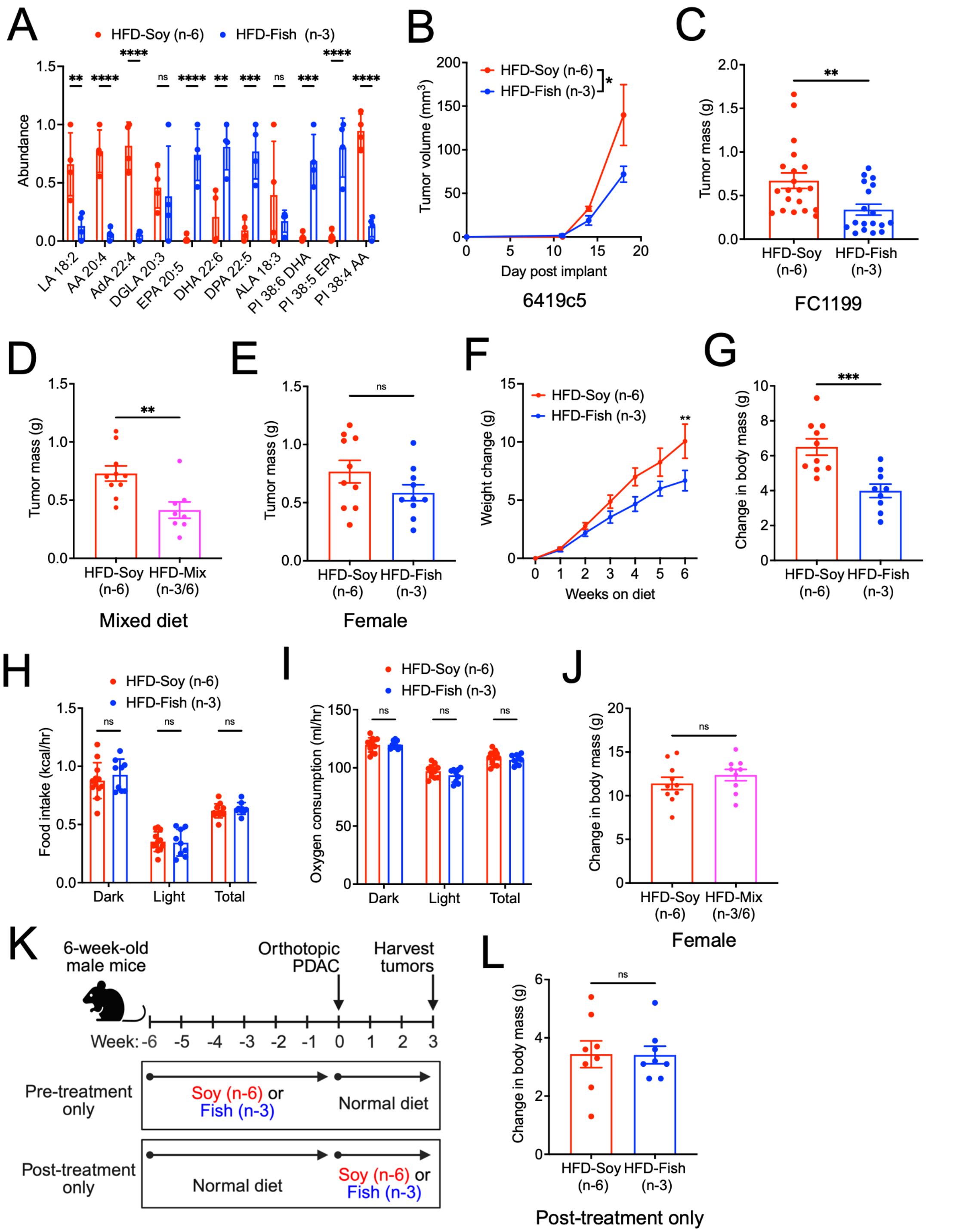

Fig. S2. Omega-3 fatty acid–mediated suppression of PDAC growth is independent of weight gain and requires continued dietary exposure during tumor progression. **(A)** MALDI-mass spectrometry imaging (MALDI-MSI) quantification of lipid species from orthotopic PDAC tumors from mice maintained on the indicated diets. **(B)** Tumor volume measured by ultrasound on days 11, 14, and 18 following orthotopic implantation of 6419c5 tumor cells in mice maintained on the indicated diets. **(C)** Endpoint tumor mass of orthotopic FC1199 tumors harvested at day 19 from mice maintained on the indicated diets. **(D)** Endpoint tumor mass of orthotopic 6419c5 tumors from mice maintained on the

indicated diets. **(E)** Endpoint tumor mass of orthotopic 6419c5 tumors from female mice maintained on the indicated diets. **(F)** Longitudinal body weight change in mice maintained on the indicated diets without tumor implantation. **(G)** Change in body weight measurements from mice maintained on the indicated diets over 6 weeks. **(H)** Food intake measured by metabolic cage analysis in mice maintained on the indicated diets for 6 weeks before measurements. **(I)** Oxygen consumption measured by metabolic cage analysis in mice maintained on the indicated diets for 6 weeks before measurements. **(J)** Change in body weight of mice after 6 weeks on diets. **(K)** Experimental schematic of dietary pre-treatment and post-treatment conditions. **(L)** Change in body weight for the 6 weeks prior to tumor implantation in the post-treatment cohorts. Representative data are shown from 2 (A,B,F,H,I,L) or 5 (C,D,E,G,J) independent experiments. Data are presented as mean ± SEM. Each dot represents one biologically independent mouse. Statistical analysis was performed using two-way ANOVA with Šídák's multiple comparisons test (A,B,F,H,I) or Welch’s t test (C,D,E,G,J,L). ns, not significant, *P < 0.05, **P < 0.01, ***P < 0.001, ****P < 0.0001.

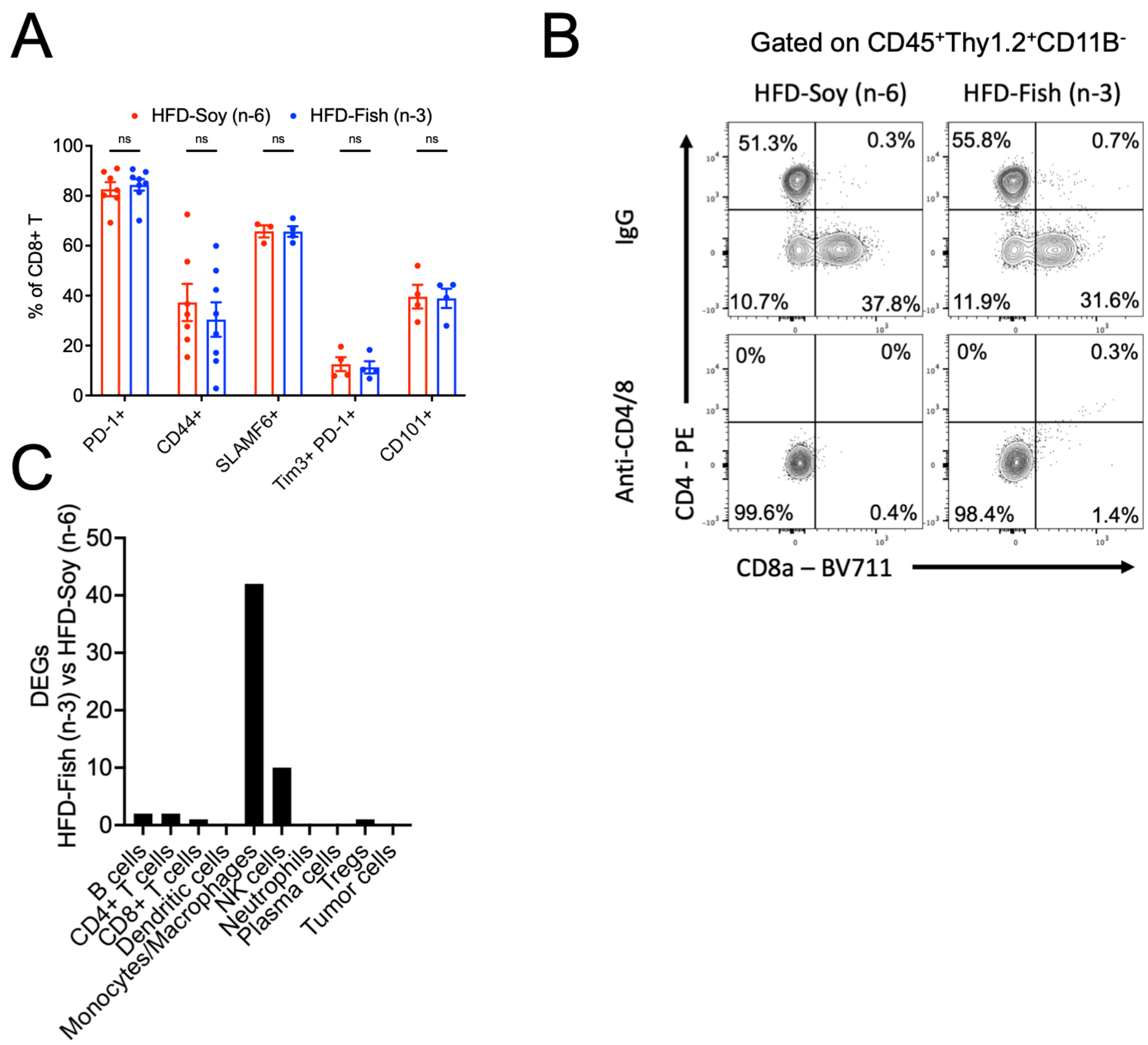

Fig. S3. T cell activation and depletion analyses in mice maintained on omega-3 and omega-6 diets. (A) Quantification of flow cytometric analysis of activation and exhaustion-associated markers on CD8$^+$ T cells isolated from orthotopic 6419c5 tumors from mice maintained on the indicated diets. Each dot represents one biologically independent mouse. **(B)** Representative flow cytometry plots of CD4$^+$ and CD8$^+$ T cells (CD45$^+$Thy1.2$^+$CD11B$^-$) from orthotopic 6419c5 tumors from mice maintained on the indicated diets following treatment with isotype (IgG) control antibodies or anti-CD4 and anti-CD8 depleting antibodies. **(C)** Quantification of differentially expressed genes between the indicated diets in the populations identified in (Fig. 2D). Representative data are shown from 2 (A, B) or 1 (C) independent experiments. Data are presented as mean ± SEM (A) or exact number (C). Statistical analysis was performed using two-way ANOVA with Šídák's multiple comparisons test (A). ns, not significant, *P < 0.05, **P < 0.01, ***P < 0.001, ****P < 0.0001.

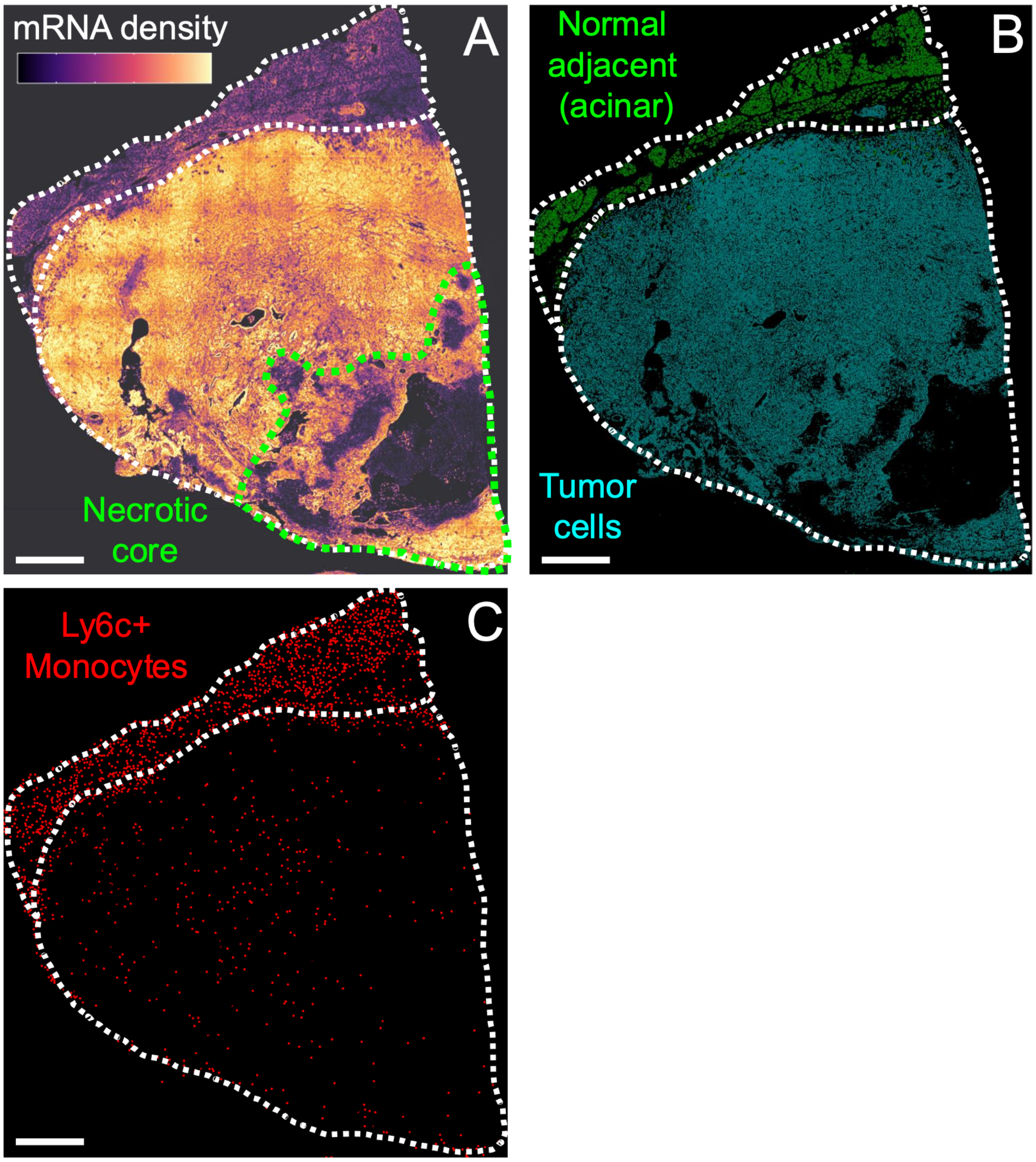

Fig. S4. Spatial localization of Ly6C⁺ monocytes and tumor-associated macrophage populations in orthotopic PDAC tumors. (A-C) Spatial transcriptomic analysis of orthotopic 6419c5 tumors from Fig. 3. **(A)** Total mRNA density map from spatial transcriptomic analysis. Green dotted line encircles area of low mRNA density specified as necrotic. **(B)** Spatial distribution of normal acinar cells (green) and tumor cells (cyan). **(C)** Spatial overlays showing localization of Ly6C⁺ monocytes. Images from n = 1 biologically independent tumor. Data are representative of 1 independent experiment. Scale bars, 1 mm.

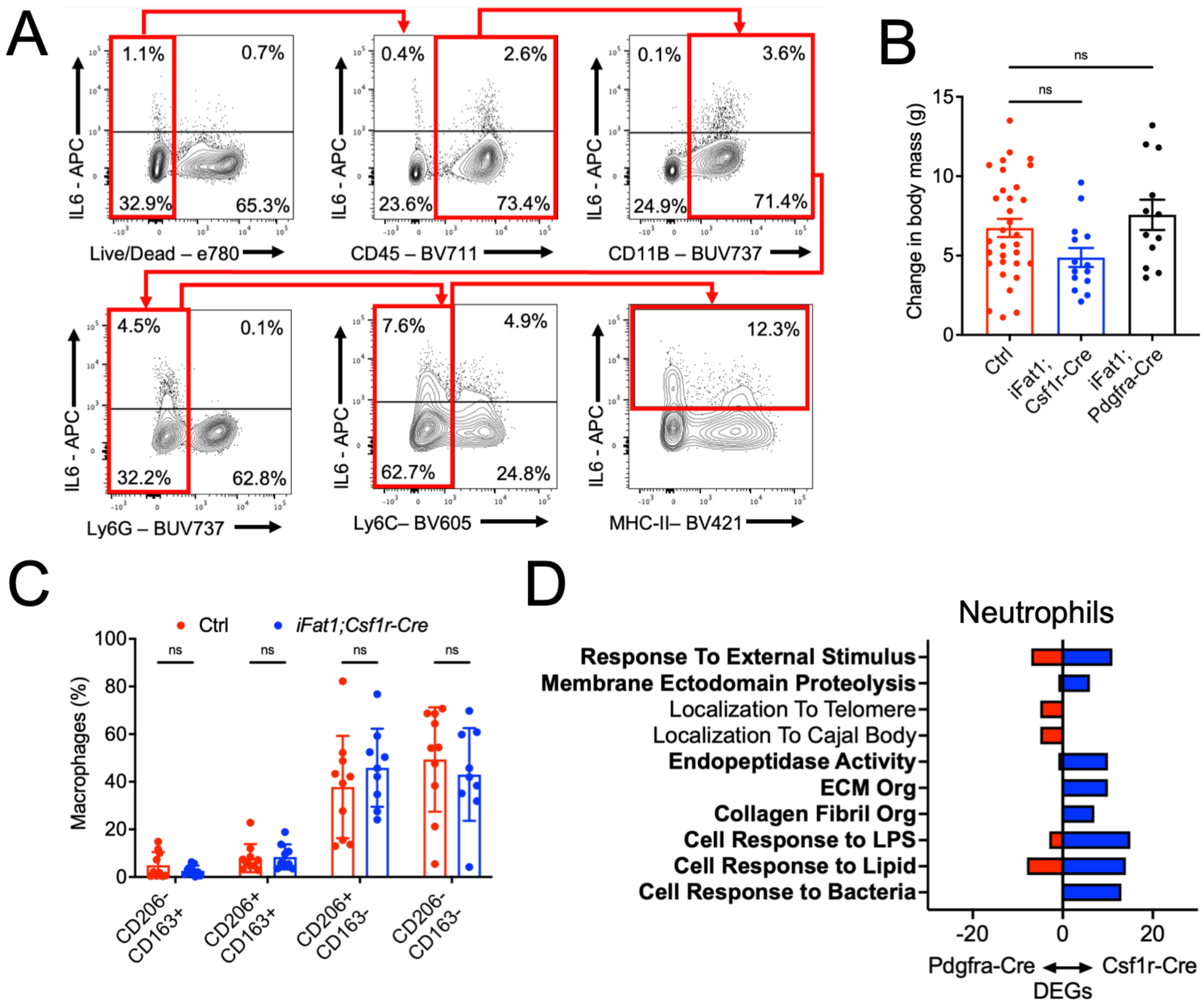

Fig. S5. Additional characterization of iFat1 mouse models and neutrophil transcriptional programs. (A) Representative flow cytometry gating of live CD45+Thy1.2-CD11b+Ly6G-Ly6C- IL-6 expressing cells from PDAC tumors following ex vivo stimulation with LPS. **(B)** Change in body weight measurements from control, *Csf1r-Cre;iFat1,* or *Pdgfra-Cre;iFat1* mice maintained on HFD-Soy (n-6) diet for 6 weeks prior to orthotopic PDAC tumor implantation. **(C)** Flow cytometric analysis of macrophage populations from orthotopic tumors of control and *Csf1r-Cre;iFat1* mice. **(D)** Differential pathway enrichment analysis of neutrophils identified by single-cell RNA sequencing in Fig. 5. Representative data are shown from 3 (A-C) or 1 (D) independent experiments. Data are presented as mean ± SEM. Each dot represents one biologically independent mouse. Statistical analysis was performed using one-way ANOVA with multiple-comparison correction (B) or two-way ANOVA with Šídák's multiple comparisons test (C). ns, not significant, *P < 0.05, **P < 0.01, ***P < 0.001, ****P < 0.0001.

Tables

Table S1. DNA-barcoded antibodies used for single-cell protein profiling

Specificity	Clone
anti-mouse TCR V gamma 1.1/Cr4	2.11
anti-mouse CD28 (Tp44)	37.51
anti-mouse CD38	90
anti-mouse CD16/32	93
anti-mouse CD45.2	104
anti-mouse CD31	390
anti-mouse TCR V gamma 3	536
anti-mouse CD317 (BST2, PDCA-1)	927
anti-mouse FceRIa	MAR-1
anti-mouse CD103	2E7
anti-mouse CD304 (Neuropilin-1)	3E12
anti-mouse CD3e	145-2C11
anti-mouse CD80	16-10A1
anti-mouse CD3	17A2
anti-mouse CD137	17B5
anti-mouse CD94	18d3
anti-mouse Ly-6G	1A8
anti-mouse CD1d (CD1.1, Ly-38)	1B1
anti-mouse P2X7R	1F11
anti-mouse TIGIT (Vstm3)	1G9
anti-mouse CD138 (Syndecan-1)	281-2
anti-mouse CD196 (CCR6)	29-2L17
anti-mouse CD335 (NKp46)	29A1.4
anti-mouse CD117 (c-kit)	2B8
anti-mouse/rat CD61	2C9.G2 (HMβ3-1)
anti-mouse/human KLRG1 (MAFA)	2F1/KLRG1
anti-mouse CD194 (CCR4)	2G12
anti-mouse CD45	30-F11
anti-mouse CD90.2	30-H12
anti-mouse Ly108	330-AJ
anti-mouse IL-21R	4A9
anti-mouse CD197 (CCR7)	4B12
anti-mouse DR3	4C12
anti-mouse CD8a	53-6.7
anti-mouse CD5	53-7.3

anti-mouse CD122 (IL-2Rbeta)	5H4
anti-mouse CD272 (BTLA)	6A6
anti-mouse CD19	6D5
anti-mouse CD160	7H1
anti-mouse CD45.1	A20
anti-mouse CD127 (IL-7Ra)	A7R34
anti-mouse TCR V alpha2	B20.1
anti-mouse TCR V alpha8.3	B21.14
anti-mouse F4/80	BM8
anti-mouse CD45RB	C363-16A
anti-human/mouse/rat CD278 (ICOS)	C398.4A
anti-mouse CD223 (LAG-3)	C9B7W
anti-mouse CD183 (CXCR3)	CXCR3-173
anti-mouse Ly-6A/E (Sca-1)	D7
anti-mouse IL-33Ralpha (IL1RL1, ST2)	DIH9
anti-mouse CD357 (GITR)	DTA-1
anti-mouse CD39	Duha59
anti-mouse CD81	Eat-2
anti-human/mouse integrin beta7	FIB504
Rat IgG1, d Isotype Ctrl	G0114F7
anti-mouse CD86	GL-1
anti-mouse TCR g/d	GL3
anti-human/mouse CD49f	GoH3
anti-mouse CD69	H1.2F3
anti-mouse TCR beta chain	H57-597
anti-mouse Ly-6C	HK1.4
anti-mouse CD48	HM48-1
anti-mouse CD270 HVEM (TR2)	HMHV-1B18
anti-mouse CD49a	HMα1
anti-mouse CD49b	HMα2
anti-mouse CD29	HMβ1-1
Armenian Hamster IgG Isotype Ctrl	HTK888
anti-mouse CD124 (IL-4Ralpha)	I015F8
anti-mouse/human CD44	IM7
anti-mouse TCR Vbeta8.1, 8.2	KJ16-133.18
anti-mouse TCR V alpha8.3	KT50
anti-mouse CD185 (CXCR5)	L138D7
anti-mouse/rat/human CD27	LG.3A10

anti-mouse CD24	M1/69
anti-mouse/human CD11b	M1/70
anti-mouse CD11a	M17/4
anti-mouse I-A/I-E	M5/114.15.2
anti-mouse CD30	mCD30.1
anti-mouse CD62L	MEL-14
anti-mouse CD178 (FasL)	MFL3
anti-mouse CD83	Michel-19
anti-mouse CD274 (B7-H1, PD-L1)	MIH6
anti-mouse VISTA (PD-1H)	MIH63
Mouse IgG2a, k isotype Ctrl	MOPC-173
Mouse IgG1, k isotype Ctrl	MOPC-21
Mouse IgG2b, k isotype Ctrl	MPC-11
anti-mouse TCR Vbeta5.1, 5.2	MR9-4
anti-mouse CD9	MZ3
anti-mouse CD11c	N418
anti-mouse CD200R (OX2R)	OX-110
anti-rat CD90/mouse CD90.1	OX-7
anti-mouse CD134 (OX-40)	OX-86
anti-mouse CD200 (OX2)	OX-90
anti-mouse CD25	PC61
anti-mouse NK-1.1	PK136
anti-mouse CD49d	R1-2
anti-mouse/human CD45R/B220	RA3-6B2
anti-mouse Ly-6G/Ly-6C (Gr-1)	RB6-8C5
anti-mouse CD55 (DAF)	RIKO-3
anti-mouse CD2	RM2-5
anti-mouse CD4	RM4-5
anti-mouse CD279 (PD-1)	RMP1-30
anti-mouse CD366 (Tim-3)	RMT3-23
Rat IgG1, k isotype Ctrl	RTK2071
Rat IgG2a, k Isotype Ctrl	RTK2758
Rat IgG2c, k Isotype Ctrl	RTK4174
Rat IgG2b, k Isotype Ctrl	RTK4530
anti-mouse CD43	S11
anti-mouse CX3CR1	SA011F11
anti-mouse CD182 (CXCR2)	SA044G4
anti-mouse CD186 (CXCR6)	SA051D1
anti-mouse CD192 (CCR2)	SA203G11

anti-mouse CD198 (CCR8)	SA214G2
anti-mouse CD20	SA275A11
anti-mouse CD95 (Fas)	SA367H8
anti-mouse CD34	SA376A4
anti-mouse CD150 (SLAM)	TC15-12F12.2
anti-mouse TER-119/Erythroid Cells	TER-119
anti-mouse Izumo1r (FR4)	TH6
anti-mouse CD226	TX42.1
anti-mouse CD155	TX56
anti-mouse CD73	TY/11.8
anti-mouse CD273 (B7-DC, PD-L2)	TY25
anti-mouse TCR V gamma 2	UC3-10A6
anti-mouse Ly.49A	YE1/48.10.6
anti-mouse CD54	YN1/1.7.4
anti-mouse CD8b	YTS156.7.7
anti-mouse CD120B	TR75-89

Table S2. Custom 10X Xenium probe panel

Gene	Ensembl ID	Probesets
Ifnb1	ENSMUSG00000048806	8
Ifne	ENSMUSG00000045364	8
Ifna1	ENSMUSG00000095498	7
Ifng	ENSMUSG00000055170	8
Il18r1	ENSMUSG00000026070	8
Il17f	ENSMUSG00000041872	8
Ifnl2	ENSMUSG00000059128	6
Il17a	ENSMUSG00000025929	8
Cd70	ENSMUSG00000019489	8
Nkx1-1	ENSMUSG00000029112	4
Fga	ENSMUSG00000028001	8
Muc5ac	ENSMUSG00000037974	8
Zp3	ENSMUSG00000004948	8
Ibsp	ENSMUSG00000029306	8
Il21	ENSMUSG00000027718	8
Plg	ENSMUSG00000059481	8
Klrg1	ENSMUSG00000030114	8
Ifnk	ENSMUSG00000042993	8
Il17c	ENSMUSG00000046108	6
Il10	ENSMUSG00000016529	8
Spp1	ENSMUSG00000029304	3
Icam4	ENSMUSG00000001014	8
Foxp3	ENSMUSG00000039521	8
Cd69	ENSMUSG00000030156	8
Xcl1	ENSMUSG00000026573	6
Cxcr3	ENSMUSG00000050232	8
Xcr1	ENSMUSG00000060509	8
Icam5	ENSMUSG00000032174	8
Gzma	ENSMUSG00000023132	8
Il4	ENSMUSG00000000869	8
Pdcd1	ENSMUSG00000026285	8
Cxcl9	ENSMUSG00000029417	8
Adam2	ENSMUSG00000022039	8
Il25	ENSMUSG00000040770	8
Il17b	ENSMUSG00000024578	6
Cxcl10	ENSMUSG00000034855	8
Gzmb	ENSMUSG00000015437	8

Itgad	ENSMUSG00000070369	8
Il6	ENSMUSG00000025746	8
Cd8a	ENSMUSG00000053977	8
Dll3	ENSMUSG00000003436	8
Ctla4	ENSMUSG00000026011	8
Dlk1	ENSMUSG00000040856	8
Clec9a	ENSMUSG00000046080	8
Cd8b1	ENSMUSG00000053044	8
Il2rb	ENSMUSG00000068227	8
Slc2a2	ENSMUSG00000027690	8
Fcgr4	ENSMUSG00000059089	8
Comp	ENSMUSG00000031849	8
Icam2	ENSMUSG00000001029	8
Ccl11	ENSMUSG00000020676	8
Sell	ENSMUSG00000026581	8
Icos	ENSMUSG00000026009	8
Il12a	ENSMUSG00000027776	8
Itgax	ENSMUSG00000030789	8
Madcam1	ENSMUSG00000020310	8
Cd3e	ENSMUSG00000032093	8
Hic1	ENSMUSG00000043099	8
Ccl20	ENSMUSG00000026166	8
Xist	ENSMUSG00000086503	3
Itga2b	ENSMUSG00000034664	8
Il2ra	ENSMUSG00000026770	8
Lamc3	ENSMUSG00000026840	8
Siglecf	ENSMUSG00000039013	8
Flt3	ENSMUSG00000042817	8
Trbc1	ENSMUSG00000076490	7
Cd80	ENSMUSG00000075122	8
Pdcd1lg2	ENSMUSG00000016498	8
Il18rap	ENSMUSG00000026068	8
Cd4	ENSMUSG00000023274	8
Notch4	ENSMUSG00000015468	8
S100b	ENSMUSG00000033208	8
Notch3	ENSMUSG00000038146	8
Aif1	ENSMUSG00000024397	3
Il12rb2	ENSMUSG00000018341	8
Itga10	ENSMUSG00000090210	8

Il2rg	ENSMUSG00000031304	8
Itgam	ENSMUSG00000030786	8
Gfap	ENSMUSG00000020932	8
Ccr5	ENSMUSG00000079227	8
Cd28	ENSMUSG00000026012	8
Itgb2	ENSMUSG00000000290	8
Cd19	ENSMUSG00000030724	8
Dusp18	ENSMUSG00000047205	8
Thy1	ENSMUSG00000032011	8
Cd86	ENSMUSG00000022901	8
Cd27	ENSMUSG00000030336	8
Ccl5	ENSMUSG00000035042	7
Ccr9	ENSMUSG00000029530	8
Slamf6	ENSMUSG00000015314	8
Icosl	ENSMUSG00000000732	8
E2f1	ENSMUSG00000027490	8
Cdc20	ENSMUSG00000006398	8
Ccr6	ENSMUSG00000040899	8
Il7r	ENSMUSG00000003882	8
Itgal	ENSMUSG00000030830	8
Il21r	ENSMUSG00000030745	8
Itgae	ENSMUSG00000005947	8
Il10ra	ENSMUSG00000032089	8
Tgfb3	ENSMUSG00000021253	8
Ccn1	ENSMUSG00000028195	8
Rspo1	ENSMUSG00000028871	8
Itgb7	ENSMUSG00000001281	8
Cd274	ENSMUSG00000016496	8
Il7	ENSMUSG00000040329	8
Lama1	ENSMUSG00000032796	8
Tcf7	ENSMUSG00000000782	7
Il17rb	ENSMUSG00000015966	8
Itga11	ENSMUSG00000032243	8
Itga7	ENSMUSG00000025348	8
Dll4	ENSMUSG00000027314	8
Cx3cr1	ENSMUSG00000052336	3
Vwf	ENSMUSG00000001930	8
Cd226	ENSMUSG00000034028	8
Ifnlr1	ENSMUSG00000062157	8

Rbfox3	ENSMUSG00000025576	8
Dll1	ENSMUSG00000014773	8
Il17re	ENSMUSG00000043088	8
Avil	ENSMUSG00000025432	8
Jam2	ENSMUSG00000053062	8
Cx3cl1	ENSMUSG00000031778	8
Icam1	ENSMUSG00000037405	8
Lamc2	ENSMUSG00000026479	8
Csf1r	ENSMUSG00000024621	4
Slc1a3	ENSMUSG00000005360	8
Il15ra	ENSMUSG00000023206	8
Id2	ENSMUSG00000020644	8
Il17ra	ENSMUSG00000002897	8
Pdgfrb	ENSMUSG00000024620	8
Cd34	ENSMUSG00000016494	8
Tgfb1	ENSMUSG00000002603	8
Itgb8	ENSMUSG00000025321	8
Ccn2	ENSMUSG00000019997	8
Runx3	ENSMUSG00000070691	8
Ifnar1	ENSMUSG00000022967	8
Mme	ENSMUSG00000027820	8
Il4ra	ENSMUSG00000030748	8
Axin2	ENSMUSG00000000142	8
Klf2	ENSMUSG00000055148	7
Jam3	ENSMUSG00000031990	8
Cxcl12	ENSMUSG00000061353	8
Adgre1	ENSMUSG00000004730	8
Flt3l	ENSMUSG00000110206	8
Itgb6	ENSMUSG00000026971	8
Itga8	ENSMUSG00000026768	8
Ifnar2	ENSMUSG00000022971	8
Itga5	ENSMUSG00000000555	8
Adam15	ENSMUSG00000028041	8
Itgb3	ENSMUSG00000020689	8
Adam12	ENSMUSG00000054555	8
Notch1	ENSMUSG00000026923	8
Itga3	ENSMUSG00000001507	8
Il10rb	ENSMUSG00000022969	8
Adam9	ENSMUSG00000031555	8

Vcam1	ENSMUSG00000027962	8
Itgb5	ENSMUSG00000022817	5
Aldh1a1	ENSMUSG00000053279	8
Il17rc	ENSMUSG00000030281	8
Il18	ENSMUSG00000039217	8
Sox9	ENSMUSG00000000567	8
Nt5e	ENSMUSG00000032420	8
Adam17	ENSMUSG00000052593	8
Fos	ENSMUSG00000021250	3
Ifngr2	ENSMUSG00000022965	8
Il6ra	ENSMUSG00000027947	8
Vim	ENSMUSG00000026728	4
Notch2	ENSMUSG00000027878	8
Itgb4	ENSMUSG00000020758	8
H2-Ab1	ENSMUSG00000073421	3
Ccl25	ENSMUSG00000023235	8
C3	ENSMUSG00000024164	8
Itga4	ENSMUSG00000027009	8
Lgr5	ENSMUSG00000020140	8
Il6st	ENSMUSG00000021756	8
Itgav	ENSMUSG00000027087	8
Tgfbr1	ENSMUSG00000007613	8
Il15	ENSMUSG00000031712	8
Mertk	ENSMUSG00000014361	8
Srebf2	ENSMUSG00000022463	8
Il13ra1	ENSMUSG00000017057	8
Mki67	ENSMUSG00000031004	8
Itga9	ENSMUSG00000039115	8
Lamb1	ENSMUSG00000002900	8
Ifngr1	ENSMUSG00000020009	8
Tgfb2	ENSMUSG00000039239	8
Adam23	ENSMUSG00000025964	8
Fn1	ENSMUSG00000026193	3
F11r	ENSMUSG00000038235	4
Vdr	ENSMUSG00000022479	8
Tnc	ENSMUSG00000028364	8
Dpp4	ENSMUSG00000035000	8
Tgfbr2	ENSMUSG00000032440	8
Klf6	ENSMUSG00000000078	8

Cd74	ENSMUSG00000024610	2
Itga2	ENSMUSG00000015533	8
Itgb1	ENSMUSG00000025809	8
Lyve1	ENSMUSG00000030787	8
Pdgfra	ENSMUSG00000029231	8
Itga6	ENSMUSG00000027111	7
Cd44	ENSMUSG00000005087	8
Apoe	ENSMUSG00000002985	3
Pecam1	ENSMUSG00000020717	8
Itga1	ENSMUSG00000042284	8
Cdh1	ENSMUSG00000000303	8
Dclk1	ENSMUSG00000027797	8
Lama2	ENSMUSG00000019899	8
Chga	ENSMUSG00000021194	8
Epcam	ENSMUSG00000045394	8
Reln	ENSMUSG00000042453	8
Ncam1	ENSMUSG00000039542	8
Ptprc	ENSMUSG00000026395	3
Chgb	ENSMUSG00000027350	8
Acta2	ENSMUSG00000035783	8
Krt8	ENSMUSG00000049382	8
Adrb1	ENSMUSG00000035283	8
Adrb2	ENSMUSG00000045730	8
Th	ENSMUSG00000000214	8
Crem	ENSMUSG00000063889	8
Scn10a	ENSMUSG00000034533	8
Tubb3	ENSMUSG00000062380	8
Entpd1	ENSMUSG00000048120	8
Havcr2	ENSMUSG00000020399	8
Cxcr6	ENSMUSG00000048521	8
Cxcr5	ENSMUSG00000047880	8
Bcl6	ENSMUSG00000022508	8
Cd101	ENSMUSG00000086564	8
Tox	ENSMUSG00000041272	8
Tbx21	ENSMUSG00000001444	8
Eomes	ENSMUSG00000032446	8
Cd38	ENSMUSG00000029084	8
Klrb1	ENSMUSG00000079299	8
Ms4a1	ENSMUSG00000024673	8

Cxcl16	ENSMUSG00000018920	3
Cxcl13	ENSMUSG00000023078	8
Ccl3	ENSMUSG00000000982	3
Ccl4	ENSMUSG00000018930	3
Chat	ENSMUSG00000021919	8
Sdc1	ENSMUSG00000020592	7
Aicda	ENSMUSG00000040627	8
Fas	ENSMUSG00000024778	8
Cd83	ENSMUSG00000015396	5
Cxcr4	ENSMUSG00000045382	8
Cd5	ENSMUSG00000024669	8
Clec10a	ENSMUSG00000000318	8
Fcer1a	ENSMUSG00000005339	8
Irf8	ENSMUSG00000041515	8
Irf4	ENSMUSG00000021356	8
Trem2	ENSMUSG00000023992	8
Cd9	ENSMUSG00000030342	7
Cd163	ENSMUSG00000008845	8
C5ar1	ENSMUSG00000049130	8
Fcgr1	ENSMUSG00000015947	8
Fcgr2b	ENSMUSG00000026656	6
Ncr1	ENSMUSG00000062524	8
Fcgr3	ENSMUSG00000059498	4
Cd200r1	ENSMUSG00000022667	8
Gata3	ENSMUSG00000015619	8
Rorc	ENSMUSG00000028150	8
Cd36	ENSMUSG00000002944	8
C1qa	ENSMUSG00000036887	3
Mrc1	ENSMUSG00000026712	6
Arg1	ENSMUSG00000019987	3
Mmp12	ENSMUSG00000049723	4
Vegfa	ENSMUSG00000023951	8
Chil3	ENSMUSG00000040809	8
Ly6c2	ENSMUSG00000022584	8
Isg15	ENSMUSG00000035692	8
Irf7	ENSMUSG00000025498	8
Nr4a1	ENSMUSG00000023034	8
Adgre5	ENSMUSG00000002885	8
Ccr7	ENSMUSG00000037944	8

Ccl22	ENSMUSG00000031779	8
Ccl17	ENSMUSG00000031780	4
Prdm1	ENSMUSG00000038151	8
Zeb1	ENSMUSG00000024238	8
Egr1	ENSMUSG00000038418	3
Actb	ENSMUSG00000029580	3
Batf	ENSMUSG00000034266	8
Bhlhe41	ENSMUSG00000030256	8
Cblb	ENSMUSG00000022637	8
Cd160	ENSMUSG00000038304	8
Cd7	ENSMUSG00000025163	8
Cited2	ENSMUSG00000039910	8
Egr2	ENSMUSG00000037868	7
Fcrl6	ENSMUSG00000070504	8
Foxo1	ENSMUSG00000044167	8
Glp1r	ENSMUSG00000024027	8
Gzmd	ENSMUSG00000059256	8
Id3	ENSMUSG00000007872	8
Ikzf2	ENSMUSG00000025997	8
Layn	ENSMUSG00000060594	8
Lef1	ENSMUSG00000027985	8
Litaf	ENSMUSG00000022500	8
Myb	ENSMUSG00000019982	8
Mybl1	ENSMUSG00000025912	8
Nectin2	ENSMUSG00000062300	8
Nfatc1	ENSMUSG00000033016	8
Nfatc2	ENSMUSG00000027544	8
Nfatc3	ENSMUSG00000031902	8
Per1	ENSMUSG00000020893	8
Ptger2	ENSMUSG00000037759	8
Rasa2	ENSMUSG00000032413	8
Rrm2	ENSMUSG00000020649	8
Runx2	ENSMUSG00000039153	8
S1pr1	ENSMUSG00000045092	8
S1pr5	ENSMUSG00000045087	8
Skil	ENSMUSG00000027660	4
Socs1	ENSMUSG00000038037	8
Spn	ENSMUSG00000051457	8
Tcf19	ENSMUSG00000050410	8

Zeb2	ENSMUSG00000026872	6
Zfp683	ENSMUSG00000049410	8
Egfr	ENSMUSG00000020122	8
Cd24a	ENSMUSG00000047139	8
Prom1	ENSMUSG00000029086	8
Axl	ENSMUSG00000002602	7
Tmem119	ENSMUSG00000054675	8
Nf1	ENSMUSG00000020716	8
Sox10	ENSMUSG00000033006	8
Olig2	ENSMUSG00000039830	8
Cdkn1b	ENSMUSG00000003031	8
Cdkn1a	ENSMUSG00000023067	5
Atf3	ENSMUSG00000026628	8
B2m	ENSMUSG00000060802	3
Cd2	ENSMUSG00000027863	8
Cd244a	ENSMUSG00000004709	8
Cd6	ENSMUSG00000024670	8
Fasl	ENSMUSG00000000817	8
Gpr183	ENSMUSG00000051212	8
Gzmk	ENSMUSG00000042385	8
Il2	ENSMUSG00000027720	8
Jun	ENSMUSG00000052684	3
Junb	ENSMUSG00000052837	3
Lag3	ENSMUSG00000030124	8
Prf1	ENSMUSG00000037202	8
Ptger4	ENSMUSG00000039942	8
Rgs1	ENSMUSG00000026358	3
Rgs2	ENSMUSG00000026360	3
Rora	ENSMUSG00000032238	8
Smad4	ENSMUSG00000024515	8
Stat4	ENSMUSG00000062939	8
Stat5a	ENSMUSG00000004043	8
Tigit	ENSMUSG00000071552	8
Tnf	ENSMUSG00000024401	8
Tnfrsf9	ENSMUSG00000028965	8
Tnfsf14	ENSMUSG00000005824	8
Top2a	ENSMUSG00000020914	8
Egf	ENSMUSG00000028017	8
Pdgfa	ENSMUSG00000025856	8

Gas6	ENSMUSG00000031451	8
Selplg	ENSMUSG00000048163	8
Ptdss1	ENSMUSG00000021518	8
Pdgfb	ENSMUSG00000000489	8
Pdgfc	ENSMUSG00000028019	8
Pdgfd	ENSMUSG00000032006	8
Acaa1a	ENSMUSG00000036138	8
Acaa1b	ENSMUSG00000010651	8
Fads1	ENSMUSG00000010663	8
Fasn	ENSMUSG00000025153	8
Scd1	ENSMUSG00000037071	8
Acot1	ENSMUSG00000072949	8
Acsl4	ENSMUSG00000031278	8
Cpt1a	ENSMUSG00000024900	8
Cpt2	ENSMUSG00000028607	8
Crat	ENSMUSG00000026853	8
Soat1	ENSMUSG00000026600	8
Acat1	ENSMUSG00000032047	8
Pex5	ENSMUSG00000005069	8
Hadha	ENSMUSG00000025745	8
Dhcr24	ENSMUSG00000034926	8
Srebf1	ENSMUSG00000020538	8
Hmgcr	ENSMUSG00000021670	8
Abca1	ENSMUSG00000015243	8
Abcg1	ENSMUSG00000024030	8
Sqle	ENSMUSG00000022351	8
Ldlr	ENSMUSG00000032193	8
Scarb1	ENSMUSG00000037936	8
Cyp27a1	ENSMUSG00000026170	8
Pparg	ENSMUSG00000000440	8
Ppara	ENSMUSG00000022383	8
Ppard	ENSMUSG00000002250	8
Nr1h3	ENSMUSG00000002108	8
Lrp1	ENSMUSG00000040249	8
Lox	ENSMUSG00000024529	8
Acss2	ENSMUSG00000027605	8
Acly	ENSMUSG00000020917	8
Ldha	ENSMUSG00000063229	4
Hk2	ENSMUSG00000000628	8

Pgam1	ENSMUSG00000011752	7
Tpi1	ENSMUSG00000023456	8
Gpx1	ENSMUSG00000063856	3
Gpx4	ENSMUSG00000075706	8
Ndufs2	ENSMUSG00000013593	8
Ndufb5	ENSMUSG00000027673	8
Ndufa10	ENSMUSG00000026260	8
Cox5a	ENSMUSG00000000088	4
Cox11	ENSMUSG00000020544	8
Sdha	ENSMUSG00000021577	8
Sdhc	ENSMUSG00000058076	8
Atp5g3	ENSMUSG00000018770	8
Atp4a	ENSMUSG00000005553	8
Dlat	ENSMUSG00000000168	8
Cs	ENSMUSG00000005683	8
Fh1	ENSMUSG00000026526	8
Pcx	ENSMUSG00000024892	8
Idh2	ENSMUSG00000030541	8
Idh1	ENSMUSG00000025950	8
Idh3b	ENSMUSG00000027406	8
Mdh1	ENSMUSG00000020321	8
Fabp4	ENSMUSG00000062515	8
Fabp5	ENSMUSG00000027533	3
Cyc1	ENSMUSG00000022551	8
Slc2a1	ENSMUSG00000028645	8
Slc7a11	ENSMUSG00000027737	8
Uqcr10	ENSMUSG00000059534	4
Suclg1	ENSMUSG00000052738	8
Pfkl	ENSMUSG00000020277	8
Adora2b	ENSMUSG00000018500	8
Agpat4	ENSMUSG00000023827	8
Agpat1	ENSMUSG00000034254	8
Ptges	ENSMUSG00000050737	8
Ptges2	ENSMUSG00000026820	8
Gsr	ENSMUSG00000031584	8
Mafa	ENSMUSG00000047591	8
Ins1	ENSMUSG00000035804	8
Ins2	ENSMUSG00000000215	8
Gcg	ENSMUSG00000000394	8

Ptgs2	ENSMUSG00000032487	4
Ptgs1	ENSMUSG00000047250	8
Alox5	ENSMUSG00000025701	8
Alox15	ENSMUSG00000018924	4
Pla2g4a	ENSMUSG00000056220	8
P2ry12	ENSMUSG00000036353	5
Slco4a1	ENSMUSG00000038963	8
Slco3a1	ENSMUSG00000025790	8
Il5	ENSMUSG00000036117	8
Il13	ENSMUSG00000020383	8
Il22	ENSMUSG00000074695	8
Ighg1	ENSMUSG00000076614	8
Ighg2b	ENSMUSG00000076613	8
Ighg2c	ENSMUSG00000076612	8
Ighg3	ENSMUSG00000076615	8
Ighm	ENSMUSG00000076617	8
Ighd	ENSMUSG00000104213	8
Igha	ENSMUSG00000095079	8
Ighe	ENSMUSG00000087642	8
Orc2	ENSMUSG00000026037	8
Cd55	ENSMUSG00000026399	8
Il1a	ENSMUSG00000027399	7
Car2	ENSMUSG00000027562	8
Tinagl1	ENSMUSG00000028776	8
C1qc	ENSMUSG00000036896	3
Parm1	ENSMUSG00000034981	8
Met	ENSMUSG00000009376	8
Snrpa1	ENSMUSG00000030512	8
Ifitm6	ENSMUSG00000059108	8
Plscr1	ENSMUSG00000032369	8
Perp	ENSMUSG00000019851	8
Bicc1	ENSMUSG00000014329	8
Hist1h2bc	ENSMUSG00000018102	8
F13a1	ENSMUSG00000039109	8
Rangap1	ENSMUSG00000022391	8
Cacnb3	ENSMUSG00000003352	8
Krt7	ENSMUSG00000023039	8
Retnlg	ENSMUSG00000022651	3
St3gal6	ENSMUSG00000022747	8

Trem1	ENSMUSG00000042265	8
Rasgrp3	ENSMUSG00000071042	8
Arap3	ENSMUSG00000024451	8
Mpeg1	ENSMUSG00000046805	6
Fam111a	ENSMUSG00000024691	8
Gpm6b	ENSMUSG00000031342	8
Igfbp5	ENSMUSG00000026185	8
Sp100	ENSMUSG00000026222	8
Rab3gap2	ENSMUSG00000039318	8
Nusap1	ENSMUSG00000027306	8
Tpx2	ENSMUSG00000027469	8
Lhfp	ENSMUSG00000048332	8
Gbp3	ENSMUSG00000028268	8
Depdc1a	ENSMUSG00000028175	8

SI References

1. Quehenberger O, Armando A, Dumlao D, Stephens DL, Dennis EA. Lipidomics analysis of essential fatty acids in macrophages. *Prostaglandins, Leukot Essent Fat Acids*. 2008;79(3-5):123-129. doi:10.1016/j.plefa.2008.09.021

2. Quehenberger O, Armando AM, Brown AH, et al. Lipidomics reveals a remarkable diversity of lipids in human plasma1 [S]. *J Lipid Res*. 2010;51(11):3299-3305. doi:10.1194/jlr.m009449

3. Shimshek DR, Kim J, Hübner MR, et al. Codon-improved Cre recombinase (iCre) expression in the mouse. *genesis*. 2002;32(1):19-26. doi:10.1002/gene.10023

4. Srinivas S, Watanabe T, Lin CS, et al. Cre reporter strains produced by targeted insertion of EYFP and ECFP into the ROSA26 locus. *BMC Dev Biol*. 2001;1(1):4. doi:10.1186/1471-213x-1-4

5. Clarke SE, Kang JX, Ma DWL. The iFat1 transgene permits conditional endogenous n-3 PUFA enrichment both in vitro and in vivo. *Transgenic Res*. 2014;23(3):489-501. doi:10.1007/s11248-014-9788-x

6. Reina-Campos M, Monell A, Ferry A, et al. Tissue-resident memory CD8 T cell diversity is spatiotemporally imprinted. *Nature*. Published online 2025:1-10. doi:10.1038/s41586-024-08466-x

7. Mina AI, LeClair RA, LeClair KB, Cohen DE, Lantier L, Banks AS. CalR: A Web-Based Analysis Tool for Indirect Calorimetry Experiments. *Cell Metab*. 2018;28(4):656-666.e1. doi:10.1016/j.cmet.2018.06.019
